# The effect of minimally invasive sacroiliac joint fusion compared to sham operation: a double-blind randomized placebo-controlled trial

**DOI:** 10.1016/j.eclinm.2024.102438

**Published:** 2024-02-01

**Authors:** Engelke Marie Randers, Paul Gerdhem, Britt Stuge, Elias Diarbakerli, Lars Nordsletten, Stephan M. Röhrl, Thomas Johan Kibsgård

**Affiliations:** a Division of Orthopaedic Surgery, Oslo University Hospital, Norway; b Institute of Clinical Medicine, University of Oslo, Norway; c Department of Clinical Science, Intervention and Technology (CLINTEC), Karolinska Institutet, Stockholm, Sweden; d Department of Surgical Sciences, Uppsala University, Sweden; e Department of Orthopaedics and Hand Surgery, Uppsala University Hospital, Sweden; f Department of Reconstructive Orthopedics, Karolinska University Hospital, Stockholm, Sweden

**Keywords:** Sacroiliac joint pain, Sacroiliac joint fusion, Sham surgery, Placebo, Orthopedic surgery

## Abstract

**Background:**

Minimally invasive fusion of the sacroiliac joint as treatment for low back pain may reduce pain and improve function compared to non-operative treatment, although clear evidence is lacking. The aim of this trial was to evaluate the effect of minimally invasive sacroiliac joint fusion compared to sham surgery on sacroiliac joint pain reduction.

**Methods:**

In this double-blind randomized sham surgery-controlled trial patients with clinical diagnosis of sacroiliac joint pain confirmed with sacroiliac joint injection were included at two university hospitals in Sweden and Norway. Patients were randomized by the operating surgeon at each site to minimally invasive sacroiliac joint fusion or sham surgery. The primary endpoint was group difference in sacroiliac joint pain on the operated side at six months postoperatively, measured by the Numeric Rating Scale (0–10). Un-blinding and primary analysis were performed when all patients had completed six months follow-up. The trial is closed for new participants and was registered at clinicaltrials.gov: NCT03507049.

**Findings:**

Between September 1st, 2018 and October 22nd, 2021, 63 patients were randomized, 32 to the surgical group, 31 to the sham group. Mean age was 45 years (range 26–63) and 59 of 63 (94%) patients were female. The mean reduction in the operated sacroiliac joint from baseline to six months postoperative was 2.6 Numeric Rating Scale points in the surgical group and 1.7 points in the sham group (mean between groups difference −1.0 points; 95% CI, −2.2 to 0.3; p = 0.13).

**Interpretation:**

This double-blind randomized controlled trial could not prove that minimally invasive fusion of the sacroiliac joint was superior to sham surgery at six months postoperative.

**Funding:**

10.13039/501100011594Sophies Minde Ortopedi supported a clinical research position for Engelke Marie Randers. Region Stockholm supported the cost for the Swedish ethical application and a clinical research appointment for Paul Gerdhem.

**Research in context**

**Evidence before this study**

Even though strong evidence is lacking, the use and popularity of minimally invasive sacroiliac joint fusion has increased the recent years. Much of the supporting literature is of low to moderate quality consisting of case studies and cohort studies, many with connections to the industry. Only two former non-blinded randomized controlled trials have been performed, comparing minimally invasive sacroiliac joint fusion to non-operative treatment, and both showed superiority of minimally invasive sacroiliac joint fusion. To what extent a placebo effect influences the results of surgery is not known.

**Added value of this study**

We are not aware of any other double-blind sham surgery-controlled randomized trials evaluating the effect of minimally invasive sacroiliac joint fusion. In our trial we could not prove that minimally invasive sacroiliac joint fusion was superior to sham surgery in the treatment of long-lasting severe sacroiliac joint pain at six months postoperatively. The sham group experienced a placebo response and the additional effect of sacroiliac joint fusion appeared to be low.

**Implications of all the available evidence**

Our trial provides new evidence regarding the effect of minimally invasive sacroiliac joint fusion in treating severe sacroiliac joint pain. With these findings, there should be discussion in the medical community whether an irreversible surgical procedure with related risks and complications is worth doing when the measured efficacy of surgery is so small, and a placebo effect might be a large part of that measured efficacy.

## Introduction

Low back pain is a major cause of disability worldwide.^1^ The sacroiliac joint is reported to be a possible cause of pain in 10–30% of patients with low back pain.^2^ Diagnosis of sacroiliac joint pain can be difficult and is recommended to be based on a combination of medical history, clinical examination, radiological imaging and sacroiliac joint injection.^3^^,^^4^ Optimal treatment for sacroiliac joint pain is a challenge. Evidence for the long-term effect of non-operative treatment is lacking.^4^ In patients recalcitrant to non-operative treatment surgical treatment might be an option.^5^

The first surgical treatment for sacroiliac joint pain consisted of open surgery with a high rate of complications and long recovery time.^6^ In the early 2000s minimal invasive surgical techniques of the sacroiliac joint were introduced. Even though strong evidence is lacking, the use and popularity of minimally invasive techniques have increased the recent years.^7^^,^^8^ For one surgical technique, over 80 000 surgeries have been performed worldwide since its introduction.^9^ Much of the extensive literature that exists in this field is of low methodological quality.^8^ Only two former randomized controlled trials (RCT), comparing sacroiliac joint fusion to non-operative treatment, have been completed, one in Europe and one in the United States.^10^, ^11^, ^12^ Both studies reported minimally invasive sacroiliac joint fusion to be more effective than non-operative treatment.^10^^,^^11^ The reported pain reduction in the non-operatively treated patients in these two RCTs was minimal and non-operative treatment seemed ineffectual.^10^^,^^11^

The measured efficacy of surgery in the surgical group in the two RCTs was substantial, however, to what extent a placebo effect may influence the results of minimally invasive joint fusion is not known.^10^^,^^11^^,^^13^ It has been suggested that sham surgery-controlled studies are justifiable when introducing new techniques, where supporting literature for efficacy is limited and the non-operative treatment is unsuccessful.^13^ Procedural interventions like surgery include high expectations and rituals, which are the main components of the placebo effect.^13^ To our knowledge no previous sham surgery-controlled RCT has evaluated the effect of sacroiliac joint fusion.

The aim of this trial was to evaluate the effect of minimally invasive sacroiliac joint fusion compared to that of sham surgery on sacroiliac joint pain reduction in a double-blind RCT.

## Methods

### Study design

This trial was an investigator-initiated, non-industry sponsored, prospective, double-blind, randomized sham surgery-controlled multicenter superiority trial. Patients were referred and evaluated for eligibility at the two study sites, Oslo University Hospital and Karolinska University Hospital. There was one unique operating surgeon at each site. Patients were randomized with 1:1 allocation in blocks of four or six to either sham surgery or minimally invasive sacroiliac joint fusion with triangular titanium implants (iFuse©, SI Bone®, California, US). The trial was reported according to the recommendations of Consolidated Standards of Reporting Trials,^14^^,^^15^ was completed in accordance with the trial protocol and all the authors vouch for the completeness and accuracy of the data. None of the authors or the principal investigators have any financial or competing interests. The trial protocol and the statistical analysis plan have been published^16^ and are available on clinicaltrials.gov (NCT03507049). No interim analysis was done.

### Ethics

The regional committee for Medical and Health Research ethics of South-East Norway (2017/1892/REK Sør-Øst A) and the Regional Ethics committee in Stockholm, Sweden (2018/1463-31) approved the trial. The trial was conducted in accordance with the principles of the Declaration of Helsinki. Oral and written informed consent was obtained from all patients.

### Participants

Patients with suspected sacroiliac joint pain were referred from general practitioners and from specialists in orthopedic surgery or rehabilitation medicine. All patients were evaluated for eligibility at the outpatient clinics of the two study centers, both public spine centers with responsibility for complex spine surgery for their regions (>3 million inhabitants) and nationwide. During the study period the two study sites were the only centers evaluating patients with severe sacroiliac joint pain eligible for surgery in Norway and in the majority of Sweden.

Patients eligible for inclusion were 21–70 years, fulfilling diagnostic inclusion criteria of severe sacroiliac joint pain lasting for more than six months or more than 18 months if pregnancy-related sacroiliac joint pain (Table 1, *Diagnosis and eligibility criteria are discussed in Section 3.1*, Supplementary appendix). All patients had a national identity number where sex is identified at birth according to the national law as female or male. Which gender patients identified as, was not registered in this trial.

**Table 1:** Eligibility criteria.

Inclusion criteria
1. Suspected SIJ pain for >6 months, or >18 months for pregnancy induced pelvic girdle pain. 2. Between 21 and 70 years old 3. Diagnosis of the SIJ as the suspected primary pain generator based on both of the following: A. Pain pointed with a single finger (Fortin Finger Test) at or close to the posterior superior iliac spine (PSIS) with possible radiation into buttocks, posterior thigh or groin B. At least 3 of 6 clinical tests for SIJ pain (3) 1. Compression 2. Posterior Pelvic Pain Provocation test—P4 3. Palpation of the long dorsal sacroiliac ligament 4. Patrick FABER’s test 5. Active Straight Leg Raise (ASLR) test 6. Gaenslen's test 4. SIJ pain of at least 5 on the Numeric Rating Scale (=NRS, where 0 is no pain at all and 10 is worst imaginable pain). 5. Oswestry Disability Index (ODI) score of at least 30%. 6. Reduced SIJ pain (Numeric Rating Scale = NRS) of at least 50% of the pre injection NRS score after fluoroscopically or computed tomography guided controlled injection of local anesthetic into the SIJ. 7. Bilateral SIJ pain, if one dominant side. If eligible the dominant painful SIJ will be treated in the study. 8. Patient should have tried adequate forms of conservative treatment with little or no response. 9. Mentally and physically able to comply with study protocol. 10. Signed study-specific informed consent.
Exclusion criteria
1. Pain due to other causes, such as lumbar disc degeneration, lumbar disc herniation, lumbar spondylolisthesis, lumbar spinal stenosis, lumbar facet degeneration, and lumbar vertebral body fracture. 2. Sacroiliac pathology caused by auto-immune disease (e.g. ankylosing spondylitis), neoplasia or crystal arthropathy. 3. History of recent (<1 year) fracture of the pelvis with documented malunion, non-union of sacrum or ilium or any type of internal fixation of the pelvic ring. 4. Spine surgery during the past 12 months. 5. Previously diagnosed or suspected osteoporosis (defined as T-score < −2.5 or history of osteoporotic fracture). 6. Documented osteomalacia or other metabolic bone disease. 7. Any condition or anatomy that makes treatment with the iFuse Implant System infeasible. 8. Patients with prior SIJ surgery.

SIJ = sacroiliac joint; FABER's test: Flexion ABdution External Rotation test; NRS = Numeric Rating Scale, where 0 is no pain, and 100 is worst possible pain.

### Randomization and masking

Computer-generated randomization stratified by site was performed by the operating surgeon (one at each site) in the operating room after the patient was under general anesthesia. Randomization was done using the Viedoc database supplied by the Clinical Trial Unit at Oslo University Hospital (Viedoc©, Viedoc Technologies®). All other parties than the operating surgeon, including follow-up assessors, were blinded as to which intervention the patient was randomized to (*section 3.1*, Supplementary appendix).

### Procedures

#### Interventions

All patients underwent general anesthesia of similar length of time. The procedure started with a three to five cm long skin incision over the posterolateral aspect of the pelvis.

#### Active intervention

Guide pins were inserted over the sacroiliac joint at the desired entry point, verified by fluoroscopy according to the surgical technique manual (iFuse©, SI Bone®).^17^ The surgeon drilled and broached over the pins, and inserted three triangular titanium implants over the sacroiliac joint.

#### Sham surgery (placebo intervention)

A blunt guide pin was inserted through the muscle to the cortical ilium and removed. The procedure was simulated as if implants were inserted.

In both groups an injection with local anesthetic of the sacroiliac joint was performed under fluoroscopic guidance. After wound closure a subcutaneous injection of local anesthetic around the wound was given. Correct implant position was confirmed with intraoperative CT for all Swedish patients and the last 14 Norwegian patients. Prior to this fluoroscopy was used in the operating room to confirm placement of implants in the first 27 patients in Norway. Images were not transferred to the hospitals radiological systems, so they were unavailable for patients, caregivers and follow-up assessors in the blinded follow-up period.

#### Data collection and follow-up

Patients completed questionnaires and functional tests at baseline, before diagnostic injection, preoperatively, postoperatively, at three months and at six months (Supplementary Table S1, Supplementary appendix). After completed six-months follow-up the patients were unblinded. All data and any adverse events were registered in the Viedoc database. Patients were allowed to crossover to minimally invasive sacroiliac joint fusion after six months if they had persistent sacroiliac joint pain, and those with bilateral pain were offered surgery of the non-operated side. All patients will be followed for two and five years postoperative.

Patients received standard treatment pre- and postoperatively with no group differences. Postoperative restrictions included partial weight-bearing on crutches for four to eight weeks which was progressively increased to full ambulation. CT scans were performed after unblinding.

On clinicaltrials.gov the sham surgery procedure was initially described as different at the two sites. After study start, the sham surgery procedures were observed on both study sites and found to be identical and therefore the description was revised on clinicaltrials.gov. Furthermore, the primary outcome for the trial lacked the wording “group difference” which was included prior to finalizing the statistical analysis plan. These changes are outlined in the published protocol.

The data was unavailable to investigators and data analysts until June 2022.

### Outcomes

The primary outcome was group difference in pain measured with the Numeric Rating Scale (NRS, range 0–10, where 0 is no pain and 10 is worst imaginable pain) in the operated sacroiliac joint at six months postoperatively.^18^ A difference of 2 NRS points or more was regarded as the target difference between groups.^19^ The target difference is the value used in sample size calculations as the difference sought to be detected in the primary outcome between the intervention groups that is considered as the smallest worthwhile effect (the smallest beneficial effect) that justifies the costs, harms and inconvenience of the intervention.^15^

Secondary outcomes included the Oswestry Disability Index ranging from 0 (minimal disability) to 100 (bedbound/full disability),^20^ the Pelvic Girdle Questionnaire ranging from 0 (no disability) to 100 (severe disability),^21^ the EQ-5D, a standardized measure of health status which describes the societal perspective on health measured by the EQ-5D-5L index that ranges from approximately 0 (worst health state) to approximately 1 (best health state),^22^ and the EQ visual analog scale 0 to 100 (where 0 is worst imaginable health status and 100 is best health status).^23^ The functional tests consisted of the 6-min walking test (distance in meters walked in 6 min),^24^ and the Timed Up and Go test (the time in seconds to raise from sitting in a chair, walk 3 m and back).^25^

Furthermore, data was collected on work status, ambulatory status, pain in the non-operated sacroiliac joint and overall pelvic girdle pain. Adverse events, including re-intervention in the operated sacroiliac joint, implant loosening, and fractures were recorded. Patient assessment of treatment, including what procedure the patient thought they had received, and patient satisfaction with treatment was collected at all follow-ups (*section 3.5*, Supplementary appendix). All assessments at three- and six-month follow-up were done by blinded investigators.

### Statistical analysis

For the sample calculation we assumed a change resulting in a mean 2 points difference between the groups for the main outcome in NRS on the operated side at six months postoperatively.^16^^,^^19^ The standard deviation was set to 2.5 points and the probability of type 1 error (alpha) to 0.05. Based on these assumptions we calculated with a two-sided test a sample size of 25 patients in each group with 80% power.^16^ Due to possible drop-out of 20%, 30 patients were planned to be included in each group, giving 60 patients in total.

Data from the intervention groups was compared based on the intention to treat (ITT) principle. Differences between two independent groups were done by the Students t-test or, in the case of non-normal distribution, the Mann-Whitney-U-test. Contingency tables were analyzed by the chi square test. The proportion of patients in both intervention groups who obtained a clinically important improvement of 2 points or more on the NRS was compared.^19^ A linear mixed model with a subject-specific random intercept and with the outcome variable at baseline, time (3 and 6 months), intervention groups and the interaction between time and intervention groups as fixed effects were used for longitudinal data^26^ (*section 3.7.1*, Supplementary appendix). We assessed the normality assumption for the primary outcome (NRS operated sacroiliac joint) as well as secondary outcomes (NRS, ODI, PGQ, EQ-5D) with histograms and boxplots and found it adequate for t-tests and linear mixed model analysis.

For the primary outcome measure, NRS for the operated sacroiliac joint at the day of the six-month follow-up was used. For the secondary outcomes the baseline value was taken from the pre-injection time point. In case of missing data at this time point, baseline data was collected from the preoperative measure point. Missing data at certain time-points reflect that this study was ongoing under the COVID-19 pandemic where COVID-regulations did not facilitate for all secondary outcomes to be gathered at all time-points. There were no missing data for the primary outcome of this trial (*section 3.7.3*, Supplementary appendix).

All statistical analyses were performed by a blinded statistician. The code for group belonging was not broken until the analyses and interpretations of the results had been completed. Data was analyzed with non-parametric tests and linear mixed models using STATA statistical software, the other analyses were performed with IBM SPSS statistical software, version 28.0.1.1.

### Role of the funding source

The funders of the trial had no role in trial design, data collection, data analysis, data interpretation, or writing of the report.

## Results

**Fig. 1:**
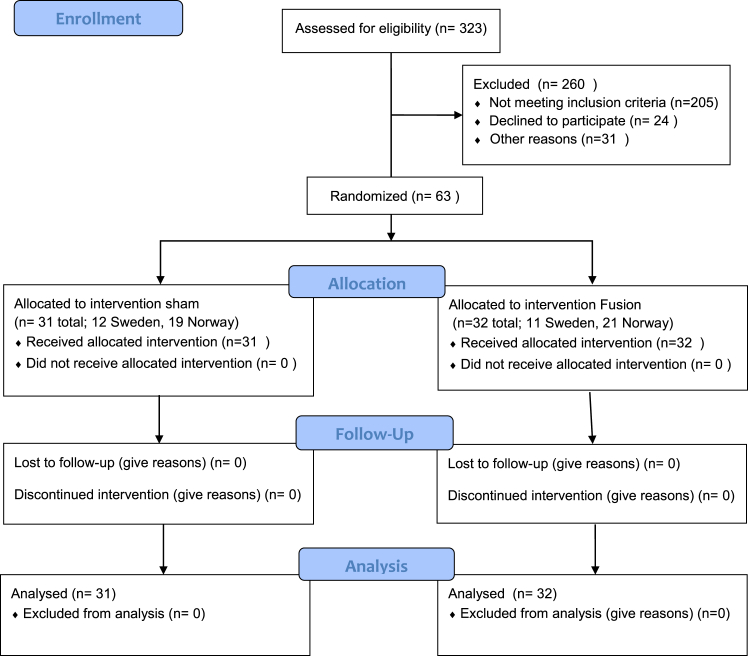
Consort flow diagram.

From September 1st, 2018 to October 22nd, 2021, a total of 63 patients were enrolled; 31 were assigned to sham surgery and 32 underwent sacroiliac joint fusion. The Norwegian site operated 40 patients (19 patients underwent sham surgery, 21 patients underwent sacroiliac joint fusion), and the Swedish site operated 23 patients (12 patients underwent sham surgery, 11 patients underwent sacroiliac joint fusion). All patients had data available at the six months follow-up (Fig. 1). The average age for both groups was 45 years (range 26–63 years), 59 of 63 patients (94%) were female, 20 patients (32%) were fully employed, and 43 patients (68%) had bilateral sacroiliac joint pain (Table 2).

**Table 2:** Patient demographics at baseline; yrs (range), n (%) or mean (95%CI).

Characteristic	Surgery group n = 32	Sham group n = 31	Both groups n = 63
Age (yrs, range)	45.3 (29–60)	44.3 (26–63)	44.8 (26–63)
Sex–Female n (%)/Male n (%)	29 (91%)/3 (9%)	30 (97%)/1 (3%)	59 (94%)/4 (7%)
Civil status—n (%)			
Married	21 (65.6%)	22 (71%)	
Partner	8 (25%)	7 (22.6%)	
Single	3 (9.4%)	2 (6.5%)	
Employment status: n (%)			
Employed	11 (34%)	9 (29%)	20 (32%)
Without paid work	6 (19%)	1 (3%)	
Partial sick leave	8 (25%)	12 (39%)	
Sick leave	7 (22%)	6 (19%)	
Partial disability leave	2 (6%)	3 (10%)	
Full disability	4 (13%)	6 (19%)	
No of Children: mean (95%CI)	2.3 (1.8–2.7)	2.6 (2.1–3.0)	
BMI: mean (95%CI)	27.7 (25.9–29.4)	26.2 (24.4–28.0)	
Prior Lumbar fusion	N = 6 (19%)	N = 3 (10%)	
Underlying cause of pain: n (%)			
In relation to pregnancy	18 (56%)	17 (53%)	
Trauma	3 (9%)	1 (3%)	
Unknown	8 (25%)	9 (28%)	
Combined	3 (9%)	4 (13%)	
Duration of symptoms: Yrs, mean (range)	11.8 (2–35)	13.1 (1,5–40)	
Previous comorbidities n (%)	17 (53%)	19 (62%)	N = 36 (58%)
Heart disease	1	0	
Hypertension	0	2	
Lung disease	1	3	
Gastrointestinal	2	0	
Depression	5	2	
Other	8	11	
Smoking n (%)	2 (6%)	0 (0%)	
Taking opioids, n (%)	7 (22%)	9 (29%)	
Unilateral/bilateral SIJ pain^a^ n (%)	9 (28%)/23(72%)	11 (36%)/20 (65%)	
Baseline:			
ODI, score, mean (95%CI)	51.2 (45.9–56.4)	52.5 (47.4–57.6)	
PGQ, score, mean (95%CI)	70.0 (64.3–75.8)	74.4 (71.2–77.6)	
NRS, score, mean (95%CI)			
Operated SIJ	7.6 (7.2–8.0)	7.7 (7.2–8.2)	
Contralateral SIJ	4.4 (3.6–5.3)	3.7 (2.7–4.6)	
Global pelvic pain	6.9 (6.4–7.5)	7.1 (6.4–7.8)	
Leg pain	5.7 (5.0–6.5)	5.8 (4.9–6.7)	
EQ-5D index, mean (95%CI)	0.63 (0.57–0.68)	0.61 (0.56–0.65)	
6 MWT, mean (95%CI)	389 (331–447)	375 (325–424)	

BMI = Body Mass Index; ODI = Oswestry Disability Index; PGQ = Pelvic girdle Questionnaire; NRS = Numeric Rating Scale; SIJ = Sacroiliac joint; EQ-5D = EuroQol 5D-5L index; 6 MWT = 6 Minute walking test.

a Bilateral SIJ pain was defined as pain in the non-operated sacroiliac joint with NRS >2.

At six months postoperative the intention-to-treat analysis showed that the difference in pain between the surgical group and the sham group was −1.0 NRS points (95%CI −2.2 to 0.3, p = 0.13) (Table 3).

**Table 3:** Outcomes presented as mean (95% confidence interval).

	Baseline		6 months		Surgery vs Sham
	Surgery	Sham	Surgery	Sham	
	Mean (95% CI)		Mean (95% CI)		(Mean difference, 95% CI, p-value)
NRS operated SIJ	**7.6** (7.2–8.0)	**7.7** (7.2–8.2)	**5.0** (4.0–5.9)	**6.0** (5.1–6.8)	**−1.0** (−2.2 to 0.3) p = 0.13
NRS global pelvic pain	**6.9** (6.4–7.5)	**7.1** (6.4–7.8)	**5.9** (5.2–6.5)	**6.3** (5.6–6.9)	**−0.5** (−1.5 to 0.5)
NRS radiating pain	**5.7** (5.0–6.5)	**5.8** (4.9–6.7)	**4.4** (3.5–5.3)	**4.8** (3.9–5.7)	−**0.4** (−1.3 to 0.6)
ODI (sum)	**51** (46–56)	**53** (47–58)	**47** (43–52)	**50** (46–54)	−**3** (−9 to 4)
PGQ (%)	**70** (64–76)	**74** (71–78)	**64** (59–69)	**68** (62–73)	−**4** (−12 to 4)
EQ-5D-5L index (range)	**0.63** (0.57–0.68)	**0.61** (0.56–0.65)	**0.65** (0.60–0.69)	**0.66** (0.61–0.70)	**−0.01** (−0.07 to 0.05)
EQ-5D VAS (Scale)	**38** (31–45)	**38** (32–45)	**40**^a^ (34–47)	**43**^a^ (37–50)	**−3** (−13 to 6)
6 MWT (meters)	**389** (331–447)	**375** (326–424)	**448**^b^ (491–405)	**435**^b^ (391–478)	**14** (−48 to 75)
TUG (s)	**10.4** (7.4–13.6)	**9.4** (8.0–13.0)	**10.6** (8.6–12.5)	**11.7** (9.8–13.7)	**−1.2**^b^ (−4.0 to 1.6)

p-value from a linear mixed model (with a subject-specific random intercept and with the outcome variable at baseline, time, intervention groups and the interaction between time and intervention groups as fixed effects).

Bold is to accentuate the mean numbers, different from the numbers in parenthesis which are the 95% confidence interval numbers.

ODI = Oswestry Disability Index; PGQ = Pelvic girdle Questionnaire; NRS = Numeric Rating Scale; SIJ = Sacroiliac joint; EQ-5D = EuroQol 5D-5L-index; EQ-5D VAS = EuroQol-5D Visual analogue scale; 6 MWT = 6 Minute walking test; TUG = Timed Up and Go test.

a N = 29 in sham group and 31 in surgery group.

b N = 30 in sham group and 30 in surgery group.

The reduction in pain in the operated sacroiliac joint in the surgical group was 2.6 NRS points (95% CI, 1.5 to 3.7) and in the sham group 1.7 NRS points (95% CI 0.6 to 2.8) from baseline to six months postoperative (Table 3). An improvement of 2 NRS points from baseline to 6 months postoperative was observed in 16 of 32 (50%) patients in the surgical group and in 13 of 31 (42%) in the sham group (Odds ratio 1.4; 95% CI 0.5 to 3.7; p = 0.52). Regarding self-reported treatment satisfaction 13 of 29 (45%) in the surgical group and 9 of 29 (31%) in the sham group reported themselves to be “much better” or “better” (Odds ratio 1.8; 95% CI 0.6 to 5.2; p = 0.27), and 11 of 29 (38%) in the surgical group and 8 of 29 (27%) in the sham group reported to be “worse” or “much worse” (Odds ratio 1.6; 95% CI 0.5 to 4.8; p = 0.40).

The physical function measured by Oswestry Disability Index, Pelvic Girdle Questionnaire, 6-min walk test and Time Up and Go test showed only small improvements from baseline to six months postoperative in both groups (Table 3).

Regarding patient blinding and the patient's ability to guess which intervention they had received, 19 of 32 (59%) patients in the surgical group and 15 of 31 (48%) in the sham group guessed the correct allocated treatment immediately postoperatively (Table 4). At the six-month follow-up the corresponding results were 15 out of 32 patients (47%) and 15 out of 31 patients (48%) (Table 4).

**Table 4:** Group allocation compared to what treatment the patients thought they received; n of total (%).

	Group allocation			
	Post-operatively		6 months	
	Surgery	Sham	Surgery	Sham
What do you think you had?				
Surgery	**19** (59%)	**14** (45%)	**15** (47%)	**7** (23%)
Sham	**11** (34%)	**15** (48%)	**12** (38%)	**15** (48%)
Don't know	**2** (6%)	**2** (6%)	**5** (16%)	**9** (29%)
Total	32 (100%)	31 (100%)	32 (100%)	31 (100%)

Bold is to accentuate the mean numbers, different from the numbers in parenthesis which are the 95% confidence interval numbers.

In the six months follow-up period seven patients out of 63 experienced eight adverse events. In the surgical group 5 patients experienced 6 adverse events; milder recurrent pain (n = 1), implant related fracture (n = 1), wound infection (n = 1), hematoma (n = 1), other (n = 2). In the sham group 2 patients experienced 2 adverse events; recurrent severe pain (n = 1) and milder recurrent pain (n = 1). None of the patients underwent additional surgery within the first 6 months. The patient with fracture was unblinded prior to six-month follow-up due to pain, another patient reported to have taken an x-ray of her pelvis during the six months follow-up period, but insisted she was not made aware of the allocated treatment. Both were included in the intention-to-treat analysis.

## Discussion

In this sham surgery-controlled RCT we could not prove that minimally invasive sacroiliac joint surgery was superior to sham surgery at six months postoperatively for the primary outcome; pain in the operated sacroiliac joint. The results were the same for all secondary outcomes.

The existing literature on minimally invasive sacroiliac joint fusion has shown larger measured efficacy of surgery in regard to pain and physical function than in our trial.^5^^,^^8^^,^^10^, ^11^, ^12^ There might be several reasons for the difference in measured efficacy of surgery, such as differences in trial populations and patient characteristics. When results from a sham surgery-controlled trial do not support existing findings, a common consideration highlighted by the surgical community is that the patients in the sham surgery-controlled trial do not represent the usual population undergoing the procedure.^13^ In this trial we used the same inclusion and exclusion criteria as the European RCT in order to get as comparable populations as possible.^10^ In the American RCT eligibility criteria excluded all patients that were in litigation, on disability leave or who were receiving workers compensation, patients that might have a poorer prognosis.^11^ These differences in patient selection due to differences in social welfare and healthcare systems between the United States of America and Europe, limits the external validity of the American RCT for use on the European population. Even when we used eligibility criteria similar to the European RCT, we still ended up with a study population with slight differences from the European RCT.^10^ When we compare our population with the European RCT the age of the included were comparable.^10^ Our trial comprised a larger proportion of women (98% vs 73%) and our patients had longer duration of symptoms (12.4 years vs 4.5 years).^10^ In both the European RCT and our RCT there were a large proportion of patients with bilateral pain and this bilateralism of symptoms could affect the measured effect of surgery when using more general outcomes such as VAS low back pain, ODI and EQ-5D.^10^ By choosing a primary end-point that takes bilateralism into consideration, bilateralism of sacroiliac joint pain should not affect the measured efficacy of surgery in our primary end-point. With the use of recognized eligibility criteria in our population we could not prove sacroiliac joint fusion to be superior to sham surgery at 6 months postoperatively. Our interpretation of this is that either the eligibility criteria used are not able to select the patients who may benefit from surgical intervention, or surgery is not as efficient as previous studies have concluded.^10^, ^11^, ^12^

The measured efficacy of surgery may be influenced by methodological quality and study bias.^8^^,^^27^ A recent systematic review intended to evaluate the methodological quality of the existing literature.^8^ This review revealed three controlled cohort studies, 35 uncontrolled studies, and only two previously published RCTs.^8^ Overall the quality of the studies were evaluated to have a GRADE rating of very low to moderate.^8^ The review found all studies except one to have some degree or high degree of bias.^8^ Many of the studies had low methodological quality and in most cases were industry-sponsored or the authors had financial ties to the industry, which might be a risk of more favorable reported outcomes.^8^^,^^28^ Industry bias in addition to other forms of bias such as group selection bias, observer bias, information bias and most importantly lack of blinding can influence and contribute to why differences in measured efficacy of minimally invasive sacroiliac joint fusion are observed.^8^^,^^29^ Our trial is an industry independent double blinded sham surgery-controlled RCT which by its design limits the risk of most of these biases.

Even though the main advantage of randomization is to generate comparable groups in order to avoid confounding, randomized trials may still be biased most commonly due to unblinded patients and observers.^29^ Both previous published RCTs hold a risk of bias due to lack of blinding.^10^^,^^11^ Procedural interventions like surgery include high expectations and rituals, and consists of both the therapeutic effect of the surgical procedure in itself, but also non-specific factors such as the patient's expectation to and experience of the procedure, including variables such as hospitalization, co-interventions and the patient-surgeon relationship.^13^^,^^30^ The placebo response consists of the same non-specific factors, the placebo effect, and the natural course of the disease.^30^ If surgery is compared to poorly founded medical treatments or even treatments that patients have experienced as ineffective, a negative expectation and a nocebo effect may arise.^31^ Thus, unblinded randomized controlled trials may compare placebo to nocebo and misleadingly conclude that the surgical procedure is effective while the outcomes may be severely biased by unspecific effects.^13^ Only a study comparing the procedure with sham surgery can test the hypothesis that the effectiveness of minimally invasive sacroiliac joint fusion is different from sham surgery.

In our study the pain reduction in the sham group was 1.7 NRS points which is a 1.2 points larger reduction than in the conservatively treated patients in the European RCT.^10^ The placebo response measured as the pain reduction observed in the sham group, consists of the placebo effect, the natural course of the disease, regression to the mean and possibly other factors not specified. In the sham group there was no additional effect from minimally invasive sacroiliac fusion. Comparable proportions in the two treatment arms reported to be “much better” or “better” in the self-reported satisfaction with treatment questionnaire. This strengthens the hypothesis that there is a large placebo response to minimally invasive sacroiliac joint fusion which influences the patient's reported pain levels. Other orthopedic sham-surgery-controlled surgical RCTs have shown similar findings to ours where sham surgery gave results comparable to the true surgical intervention.^32^, ^33^, ^34^

A possible limitation for a double-blind sham surgery-controlled RCT is that the two interventions given are easily discernible for the included patients.^29^ About half of the patients in both groups believed they had undergone minimally invasive sacroiliac joint fusion immediately postoperatively. At six months postoperatively half of the patients in both groups guessed the correct allocated treatment, whilst the other half either thought they had undergone the opposite treatment or were unsure of which treatment they were allocated to. In the sham group a larger proportion of patients were uncertain in regard to group allocation, which corresponds with their higher degree of self-reported unchanged clinical outcome in the self-reported satisfaction questionnaire. The inability of 50% of patients in each group to identify which treatment they had undergone reflects that the study-group assignments were concealed effectively.

A length of blinded follow-up of six months could be considered a possible limitation. Six months postoperative was the chosen primary end-point in the two, former completed RCTs.^10^^,^^11^ We found it important to have an endpoint comparable to existing literature. There is also an ethical aspect to holding this patient population blinded for longer than six months. The placebo response is shown to persist throughout the blinded follow-up period, regardless of length, and would not have prohibited a longer blinded follow-up period.^30^ The choice of length of follow-up must take into consideration both the benefit in evaluating the effect of the surgical procedure, but also the possible harm the study population is exposed to through receiving sham surgery and by delaying a possibly effective surgical procedure.^13^ Furthermore, the study procedure is named minimally invasive sacroiliac joint fusion, and this would imply users to believe fusion over the sacroiliac joint is expected to occur and be the reason for clinical success. The implants used are designed and proven to give immediate mechanical fixation with sacroiliac joint stabilization, followed by secondary biological fixation through osseointegration of the implants into surrounding bone.^35^ Studies have shown radiological findings of bony overgrowth across the sacroiliac joint as a sign of fusion after two years, but at what exact stage this fusion occurs is unknown.^36^ It has been shown that the clinical outcomes after minimally invasive sacroiliac joint fusion are sustained from six months postoperative without further larger improvements within the first five years.^3^ A solid fusion across the joint does not seem to add to the improvement originally obtained in clinical outcomes with this procedure, and would not justify a longer length of blinded follow-up. Therefore, we found a length of blinded follow-up of six months to be adequate and the ethical choice for this sham surgery-controlled RCT.

In conclusion, in this sham surgery-controlled RCT we could not prove that minimally invasive sacroiliac joint fusion was superior to sham surgery in the treatment of long-lasting severe sacroiliac joint pain at six months postoperatively. We could not prove that minimally invasive sacroiliac joint fusion was equal to sham surgery because this trial was not powered to address this research question. The sham group experienced a placebo response and the additional effect of sacroiliac joint fusion appeared to be low. With these findings, there should be discussion in the medical community whether an irreversible surgical procedure with related risks and complications are worth doing when the measured efficacy of surgery is so small, and a placebo effect might be a large part of that measured efficacy.

## Contributors

All authors have read and approved the final version of the manuscript.

Engelke Marie Randers and Thomas Johan Kibsgård verified the underlying data.

Engelke Marie Randers: Conceptualization, data curation, formal analysis, funding acquisition, investigation, methodology, project administration, supervision, resources, software, validation, visualisation, writing-original draft, review and editing.

Thomas Johan Kibsgård: Conceptualization, data curation, formal analysis, funding acquisition, investigation, methodology, project administration, resources, software, supervision, validation, visualisation, writing–review and editing.

Paul Gerdhem: Conceptualization, data curation, funding acquisition, investigation, methodology, project administration, resources, software, supervision, validation, visualisation, writing–review and editing.

Britt Stuge: Conceptualization, methodology, validation, supervision, writing–review and editing.

Elias Diarbakerli: Data curation, methodology, validation, supervision, writing–review and editing.

Lars Nordsletten: Methodology, supervision, validation, writing–review and editing.

Stephan M. Rörhl: Methodology, supervision, validation, writing–review and editing.

## Data sharing statement

Raw trial data from this clinical trial is not available for data sharing due to Norwegian and Swedish law regulations. The study protocol and statistical analysis plan have been published and are available at clinicaltrials.gov (NCT03507049).

## Declaration of interests

Paul Gerdhem has received lecture fees from DePuySynthes paid to him.

Thomas Johan Kibsgård has received consulting fees from Stryker, and support covering travel costs for attending spinal deformity meetings through SMAIO and SI-BONE.

Stephan M. Röhrl is on the advisory board of the Norwegian arthroplasty registry and vice president of the International RSA society. All other authors declare no competing interests.

## Appendix ASupplementary data

Supplementary materials

SIFSO Supplement Protocols and SAP SIFSO
