# Supplementary material for: The effect of minimally invasive sacroiliac joint fusion compared to sham operation: a double-blind randomized placebo-controlled trial
Source: eClinicalMedicine. 2024 Feb 1;68:102438. doi: 10.1016/j.eclinm.2024.102438 (PMC10847054; doi:10.1016/j.eclinm.2024.102438)
Supplement: Supplementary materials [file mmc1.docx]

**Supplementary material**

Table of contents

[**1.** **The SIFSO study group** 1](#_Toc150678360)

[**1.1** **Composition of study group** 1](#_Toc150678361)

[**1.2** **Funding** 2](#_Toc150678362)

[**2.** **Patient representation and involvement** 2](#_Toc150678363)

[**3.** **Supplement to Methods:** 2](#_Toc150678364)

[***3.1*** **Diagnosis of sacroiliac joint pain and eligibility criteria** 2](#_Toc150678365)

[**3.1.1** **Diagnosis of sacroiliac joint pain** 2](#_Toc150678366)

[**3.1.2** **Inclusion and exclusion criteria** 2](#_Toc150678367)

[***3.2*** **Blinding/Unblinding** 3](#_Toc150678368)

[**3.2.1** **Blinding:** 3](#_Toc150678369)

[**3.2.2** **Unblinding:** 3](#_Toc150678370)

[**3.3** **Interventions** 3](#_Toc150678371)

[**3.3.1** **Active intervention** 3](#_Toc150678372)

[**3.4** **Postoperative follow-up** 3](#_Toc150678373)

[**3.5** **Imaging** 3](#_Toc150678374)

[**3.6** **Outcome measures** 3](#_Toc150678375)

[**3.6.1** **Patient-reported outcome measurements** 3](#_Toc150678376)

[**3.6.1.1** **Numeric rating scale as primary outcome** 3](#_Toc150678377)

[**3.6.1.2** **Numeric Rating Scale (NRS)** 3](#_Toc150678378)

[**3.6.1.3** **Oswestry Disability Index (ODI)** 4](#_Toc150678379)

[**3.6.1.4** **Pelvic Girdle Questionnaire (PGQ)** 4](#_Toc150678380)

[**3.6.1.5** **The Euroqol 5 Dimension (EQ-5D-5L)** 4](#_Toc150678381)

[**3.6.2** **Functional tests** 4](#_Toc150678382)

[**3.6.2.1** **6 minute walking test (6MWT)** 4](#_Toc150678383)

[**3.6.2.2** **Timed Up and Go (TUG)** 4](#_Toc150678384)

[**3.7** **Statistical analysis** 4](#_Toc150678385)

[**3.7.1** **Mixed model** 4](#_Toc150678386)

[***3.7.2*** **Within and between participant variance** 4](#_Toc150678387)

[***3.7.3*** **Management of missing data** 4](#_Toc150678388)

[***3.7.4*** **Baseline parameters:** 5](#_Toc150678389)

[**3.7.4.1** **NRS operated sacroiliac joint outcome:** 5](#_Toc150678390)

[**4.** **Supplementary Figures and tables:** 5](#_Toc150678391)

[References 6](#_Toc150678392)

[**Table S1: Timeline of outcome measures** 8](#_Toc150678393)

[**Table S2: Missing data** 9](#_Toc150678394)

[**Table S3: Outcomes presented as mean (95% confidence interval or median, interquartile range). P-values from Students t-test or Mann-Whitney U-test.** 10](#_Toc150678395)

[**Table S4: Outcomes presented as mean (95% confidence interval) with p-values from a linear mixed model (with a subject-specific random intercept and with the outcome variable at baseline, time, intervention groups and the interaction between time and intervention groups as fixed effects).** 11](#_Toc150678396)

## **The SIFSO study group**

### **Composition of study group**

Thomas Johan Kibsgård (TJK) and Jon Dahl (JD) conceived of the study.

TJK, JD, Paul Gerdhem (PG), Engelke Marie Randers (EMR), Andreas Westberg and Britt Stuge (BS) initiated the study design and Elias Diarbakerli (ED), Lars Nordsletten and Stephan Röhrl contributed to implementation.

TJK, PG, Per Näsman at the Royal School of Technology, Stockholm, and a local statistician (Corina Silvia Rueegg) from the University of Oslo provided statistical expertise in clinical trial design. TJK and EMR and statistician Are Pripp at University of Oslo conducted the primary statistical analysis.

All authors contributed to refinement of the study protocol.

The first author drafted the final manuscript which was approved by all authors^1^.

### **Funding**

The funding source had no role in the design of this trial and no role in its excecution, analyses, interpretation of data or decisions to submit results.

Funding sources:

- Sophies Minde AS (an official grant) gave support for a clinical research position for ER.
- Region Stockholm supported the cost for the Swedish ethical application.
- PG was supported by Region Stockholm in a clinical research appointment.
- Karolinska university hospital, Stockholm, Sweden facilitated for the Swedish arm of the trial.

Trial sponsor: Oslo University Hospital

Sponsors reference: OUS ForPro nr:2017/7671

Contact name: Thomas Johan Kibsgård

Adress: Department of Orthopedic Surgery, Rikshospitalet, Oslo University Hospital, Postbox 4950 Nydalen, 0424 Oslo

Telephone: +4723076071

E-mail: [**uxkibt@ous-hf.no**](mailto:uxkibt@ous-hf.no)

## **Patient representation and involvement**

The trial had a patient representative involved, Astrid Lunestad, who is affiliated to The National society for female pelvic health («Landsforeningen kvinnelig bekkenleddhelse »(LKB)). Astrid has for many years been an active board member in LKB. She contributed in deciding the research question, both in regards to whether this trial would be of importance for this patient group and also whether it would be feasible to complete. She has contributed in producing our information pamphlet for study patients to ensure that the language used was understandable and descriptive. Furthermore, she contributed in distributing knowledge about the surgical work done at Rikshospitalet as well as promoting the study amongst patients with SIJ pain. Astrid also participated in formulating the conclusion of the RCT as well as implementing the knowledge we find to the involved patient group.

## **Supplement to Methods:**

### **Diagnosis of sacroiliac joint pain and eligibility criteria**

#### **Diagnosis of sacroiliac joint pain**

Diagnosis of sacroiliac joint pain is difficult and complex^1-3^. There is, however, some consensus and the inclusion and exclusion criteria used in this trial are based upon this consensus as well as the eligibility criteria used in the former two RCTs performed in this field^2, 4, 5^.

#### **Inclusion and exclusion criteria**

- - - 1. For inclusion and exclusion criteria see Table S1
      2. Patients in this trial were included and excluded in accordance with the eligibility criteria. Patients underwent thorough medical evaluation as well as diagnostic imaging to reveal other causes as explanation for their low back pain.
      3. Diagnostic imaging

Diagnostic imaging in the diagnostics of sacroiliac joint pain is primarily used to exclude other causes of low back pain^3^. The literature shows that there is little correspondence between degenerative and other findings in the sacroiliac joint on CT and MR and the presence of sacroiliac joint pain^6, 7^. Therefore diagnostic imaging cannot be used to determine that the pain arises from the sacroiliac joint, but rather for excluding other causes for low back pain^3^.

Causes of low back pain that can be excluded by diagnostic imaging includes spinal stenosis, degenerative lumbar spine disease, degenerative lumbar disc disease, nerve root compression as well as rheumatological causes of sacroiliac joint pain represented by f.ex. sacroiliitis on MRI.

All patients underwent MRI of the lumbosacral spine prior to inclusion into the trial.

Patient history was examined to make sure there were no underlying rheumatological cause in either blood samples or in their medical history as this also was a cause for exclusion.

### **Blinding/Unblinding**

- - 1. **Blinding:**

The only staff aware of which treatment the patient received was the operating surgeon and assisting operating staff. All other personnel were blinded as to which intervention the patients received. This included trial patients, care providers, investigators, follow-up assessors and data analysts. Only the operating surgeon had access to the patient randomization prior to completed follow-up.

- - 1. **Unblinding:**
       1. Unblinding took place in accordance with the protocol after completed 6 months follow-up. At this point both blinded investigator and patients were unblinded.
       2. Unblinding of patients outside of standard protocol took place if there was suspicion of a severe adverse event in which knowledge of implant positioning was vital for patient management.

### **Interventions**

#### **Active intervention**

The surgical manual for insertion of triangular titanium implants was followed (iFuse©, SI Bone®)^8^. Three implants were placed across the sacroiliac joint as described by the manufacturer in their surgical manual using the manufacturers standardized surgical technique.

### **Postoperative follow-up**

All patients were advised to adopt partial weight-bearing on crutches for the first 4-8 weeks postoperatively, and then gradually increasing to fully ambulatory. After 12 weeks there were no restrictions.

### **Imaging**

CT scans of the SIJ was performed preoperatively and at 6, 12, and 24 months follow-up to evaluate proper implant positioning and lack of radiologic signs of loosening.

### **Outcome measures**

All assessments at 3- and 6-months follow-up were performed by blinded investigators who were qualified health personnel. After 6 months patients and investigators were un-blinded, but follow-up will be continued for 5 years after the operation. The outcomes and the timeline for assessment is outlined in Table S1.

#### **Patient-reported outcome measurements**

#### **Numeric rating scale as primary outcome**

NRS is used as a primary outcome in previous studies on minimally invasive sacroiliac joint fusion and will make it possible to compare our results with existing literature. NRS on the operated side was chosen as primary outcome to examine the effect of the dominant pain of the patient. Furthermore, the measurement of NRS on the operated side would not be affected by bilaterality of sacroiliac joint symptoms. NRS is further shown to be more precise and valid, compared to e.g. Visual Analogue Scale (VAS) in chronic pain patients^9^. A functional outcome such as Oswestry Disability Index (ODI) was chosen as a secondary outcome because a function scale does not necessarily capture the reduced pain from the operated side in patients with long-lasting pain.

#### **Numeric Rating Scale (NRS)**

Participants are asked to classify pain intensity on the Numeric rating scale where 0 is no pain and 10 is worst imaginable pain (NRS 0-10 points). NRS is easy to administer, both verbally and graphically, and is validated as a measure of pain intensity in chronic pain patients^10^.

NRS general pelvic pain was measured and regarded as being more comparable to VAS low back pain. However, this measurement could be influenced by presence of bilateral symptoms in the patients. NRS leg pain was measured to cover the referred leg pain often seen in this patient group.

#### **Oswestry Disability Index (ODI)**

ODI was originally validated as a functional measure for LBP, and it has been validated as a measure of change in SIJ health^11^. The ODI, version 2.0, covers 1 item on pain and 9 items on activities of daily life (personal care, lifting, walking, sitting, standing, sleeping, sex life, social life and travelling)^12^. Each item is measured on a 6-point ordinal scale, ranging from best scenario to worst scenario. A percentage is calculated to get at total score between 0 (minimal disability) to 100 (maximum disability/bedbound)^12^.

#### **Pelvic Girdle Questionnaire (PGQ)**

The PGQ was developed as a condition-specific, patient-reported outcome measure for participants suffering from pelvic girdle pain^13^. It is proven to have high validity and reliability in patients with SIJ pain both during pregnancy and postpartum^14, 15^. The PGQ consists of 20 activity items and 5 symptom items on a 4-point scale (0-3)^14^. The total PGQ score is given as a percentage score ranging from 0 (no disability) to 100 (severe disability)^14^.

#### **The Euroqol 5 Dimension (EQ-5D-5L)**

The EQ-5D-5L is a standardised measure of health status reflecting the societal view on health^(16)^. The EQ-5D-5L consist of the EQ-5D-5L descriptive and the EQ-5D visual analogue scale (EQ-VAS)^(17)^. The EQ-5D-5L comprises five dimensions: mobility, self-care, usual activities, pain/discomfort and anxiety/depression^(17)^. Each dimension has 5 levels ranging from no problems to extreme problems^(17)^. The digits from the 5 dimensions can be expressed as a 5-digit number that describes the patient´s health status^(17)^. This 5-digit number can be expressed as an EQ-5D index value (range 0 to 1, where 0 = health status equal to death and 1 = best imaginable health status) by using a standardized value set. Minimum and maximum values that can be obtained however depends on the value set used. In this study the Swedish value set was used^17, 18^.

#### **Functional tests**

#### **6 minute walking test (6MWT)**

The 6MWT is used to assess a patient’s functional capacity. The 6MWT is self-paced, and involves measuring the distance a patient can walk on a level course in 6 minutes^19^.

#### **Timed Up and Go (TUG)**

Timed up and go test (TUG) is a simple test to assess a person’s mobility. It uses the time it takes for a person to rise from a chair, walk three meters, turn around, walk back to the chair and sit down^20^.

### **Statistical analysis**

#### **Mixed model**

- In the mixed model time was parametrized as a factor (dummy variable) at baseline, 3 months and 6 months.

### **Within and between participant variance**

- Within participant variance was 2.3 NRS points.
- Between participant variance was 1.4 NRS points.
  - 1. **Management of missing data:**
- Missing data were handled by pair-wise deletion.
- Data missing is demonstrated in table S2.
- No data for primary endpoint NRS operated SIJ at 6 months postoperatively was missing.
- Data was classified as missing due to inability to perform test, missing due to COVID-19 pandemic preventing hospital visit,or truly missing.
- There is 1 patient missing 3 month data in both the surgical group and the sham group. This was due to the ongoing COVID pandemic disturbing the ability for the trial team to do follow-up due to the pandemic, and due to difficulty reaching the patients within a time frame of 3 months postoperatively.
- Time Up and Go (TUG) test and 6 minute walking test (6MWT) are missing for 4-5 patients at 3 and 6 months follow-up (table S2). This was in majority due to the COVID-19-pandemic preventing patients from coming to the hospital for examination, but also because some patients were unable to complete the task due to pain.
- The patients who were unable to complete the 6MWT had 0 metres registered for their test. However, those who were unable to complete the TUG test were registered as missing as any other value would be misleading for the time-result of the test when statistical tests were done.

### **Baseline parameters:**

### **NRS operated sacroiliac joint outcome:**

- The NRS operated sacroiliac joint outcome was initially planned in the SAP to be retrieved from the preinjection assessment point. This was because some Swedish patients lacked their baseline data as they first came for evaluation at the preinjection point. Mainly due to travel regulations during the COVID-19 pandemic. As these patients had not been evaluated before, the study group decided that the preinjection data was used as baseline where baseline data was not available for NRS operated sacroiliac joint outcome.

Therefore, the study group concluded to alter the baseline parameter with which the 6 months postoperative NRS operated sacroiliac joint parameter would be compared with to the baseline assessment point so this would reflect the first assessment for all patients.

## **Supplementary Figures and tables:**

- 1. **Table S1:** Outcomes and Timeline for assessments
  2. **Table S2:** Missing data
  3. **Table S3:** Table of outcomes including 3 months results presented as mean (95% CI or median, interquartile range). P-value from Student t-test or Mann-Whitney U-test.
  4. **Table S4:** Table of outcomes including 3 months results presented as mean (95% confidence interval) with p-values from a linear mixed model (with a subject-specific random intercept and with the outcome variable at baseline, time, intervention groups and the interaction between time and intervention groups as fixed effects).
  5. Protocol: Full protocol and SAP available at clincatrials.gov NCT03507049

## References

1. Falowski S, Sayed D, Pope J, Patterson D, Fishman M, Gupta M, et al. A Review and Algorithm in the Diagnosis and Treatment of Sacroiliac Joint Pain. J Pain Res. 2020;13:3337-48.

2. Vleeming A, Albert HB, Ostgaard HC, Sturesson B, Stuge B. European guidelines for the diagnosis and treatment of pelvic girdle pain. EurSpine J. 2008;17(6):794-819.

3. Thawrani DP, Agabegi SS, Asghar F. Diagnosing Sacroiliac Joint Pain. J Am Acad Orthop Surg. 2019;27(3):85-93.

4. Sturesson B, Dengler J, Kools D, Pflugmacher R, Prestamburgo D. Sacroiliac minimal invasive fusion compared to physical therapy: six-month outcome from a multicentre randomised controlled trial. The Spine Journal. 2016;16(4):S73-S4.

5. Polly DW, Cher D, Wine K et al. Randomized Controlled Trial of Minimally Invasive Sacroiliac Joint Fusion Using Triangular Titanium Implants vs. NonSurgical Management for Sacroiliac Joint Dysfunction. Global Spine Journal. 2016;6(S 01):GP113.

6. Elgafy H, Semaan HB, Ebraheim NA, Coombs RJ. Computed tomography findings in patients with sacroiliac pain. ClinOrthopRelat Res. 2001(382):112-8.

7. Shibata Y, Shirai Y, Miyamoto M. The aging process in the sacroiliac joint: helical computed tomography analysis. J OrthopSci. 2002;7(1):12-8.

8. SI BONE. iFuse surgical technique 2020 [Available from: <https://si-bone.com/providers/solutions/ifuse/surgical-technique>.

9. Farrar JT, Young JP, LaMoreaux L, Werth JL, Poole RM. Clinical importance of changes in chronic pain intensity measured on an 11-point numerical pain rating scale. Pain. 2001;94(2):149-58.

10. Hawker GA, Mian S, Kendzerska T, French M. Measures of adult pain: Visual Analog Scale for Pain (VAS Pain), Numeric Rating Scale for Pain (NRS Pain), McGill Pain Questionnaire (MPQ), Short‐Form McGill Pain Questionnaire (SF‐MPQ), Chronic Pain Grade Scale (CPGS), Short Form‐36 Bodily Pain Scale (SF‐36 BPS), and Measure of Intermittent and Constant Osteoarthritis Pain (ICOAP). Arthritis Care Res (Hoboken). 2011;63(S11):S240-S52.

11. Copay AG, Daniel JC. Is the Oswestry Disability Index a valid measure of response to sacroiliac joint treatment? Qual Life Res. 2016;25(2):283-92.

12. Smeets R, Koke A, Lin C-WC, Ferreira ML, Demoulin C. Measures of Function in Low Back Pain/Disorders Low Back Pain Rating Scale (LBPRS), Oswestry Disability Index (ODI), Progressive Isoinertial Lifting Evaluation (PILE), Quebec Back Pain Disability Scale (QBPDS), and Roland-Morris Disability Questionnaire (RDQ). Arthritis Care Res (Hoboken). 2011;63(S11):S158-S73.

13. Stuge B, Garratt A, Krogstad JH, Grotle M. The pelvic girdle questionnaire: a condition-specific instrument for assessing activity limitations and symptoms in people with pelvic girdle pain. PhysTher. 2011;91(7):1096-108.

14. Stuge B, Krogstad Jenssen H, Grotle M. The Pelvic Girdle Questionnaire: Responsiveness and Minimal Important Change in Women With Pregnancy-related Pelvic Girdle Pain, Low Back Pain, or Both. Phys Ther. 2017;97(11):1103-13.

15. Stuge B, Jenssen HK, Grotle M. The Pelvic Girdle Questionnaire: Responsiveness and Minimal Important Change in Women With Pregnancy-Related Pelvic Girdle Pain, Low Back Pain, or Both. Phys Ther. 2017;97(11):1103-13.

16. EuroQol Foundation. EQ-5D-5L User Guide - Basic information on how to use the EQ-5D-5L instrument 2019 [updated september 2019. Available from: <https://euroqol.org/wp-content/uploads/2019/09/EQ-5D-5L-English-User-Guide_version-3.0-Sept-2019-secured.pdf>.

17. Foundation ER. EG-5D-5L About 2017 [Available from: <https://euroqol.org/eq-5d-instruments/eq-5d-5l-about/>.

18. Burström K, Teni FS, Gerdtham U-G et al. Experience-Based Swedish TTO and VAS Value Sets for EQ-5D-5L Health States. Pharmacoeconomics. 2020;38(8):839-56.

19. Chetta A, Zanini A, Pisi G et al. Reference values for the 6-min walk test in healthy subjects 20–50 years old. Respir Med. 2006;100(9):1573-8.

20. Evensen NM, Kvale A, Braekken IH. Reliability of the Timed Up and Go test and Ten-Metre Timed Walk Test in Pregnant Women with Pelvic Girdle Pain. Physiother Res Int. 2015;20(3):158-65.

| Endpoint/outcome | Baseline | Preoperative | Postoperative | 3 months | 6 months | 12 months | 24 months |
| --- | --- | --- | --- | --- | --- | --- | --- |
| NRS operated SIJ/side | x | x | x | x | x | x | x |
| Global NRS | x | x | x | x | x | x | x |
| NRS non-operated SIJ/side | x | x | x | x | x | x | x |
| Leg pain NRS | x | x | x | x | x | x | x |
| ODI | x | x |  | x | x | x | x |
| PGQ | x | x |  | x | x | x | x |
| EQ-5D-5L, including EQ-VAS scale | x | x |  | x | x | x | x |
| Clinical tests:   - Patrick´s test (FABER) - Posterior Pelvic Pain Provocation test (P4) - Gaenslens test - Mennells test - Distraction - Compression - Palpation of the long dorsal ligament and sacrotuberal ligament | x | x |  | x | x | x | x |
| Functional tests   - ASLR - ASLR range test - 6MWT - TUG | x | x |  | x | x | x | x |
| Ambulatory and work status | x | x |  | x | x | x | x |
| Adverse Events (including device breakage, migration, loosening etc) |  |  | x | x | x | x | x |
| Re-intervention of target SIJ |  |  |  | x | x | x | x |
| Patient satisfaction with treatment |  |  |  | x | x | x | x |
| Patients assessment of treatment |  |  |  | x | x | x | x |

#### **Table S1: Timeline of outcome measures**

**Footnotes table S2:**

Abbreviations and explanations: **SIJ** = Sacroiliac joint. **NRS** = Numeric Rating Scale (measured on scale 0-10 where 0 is no pain and 10 is worst imaginable pain). **ODI** = Oswestry Disability Index (scale 0-100, where 0 is normal function,100 is bedridden). **PGQ** = Pelvic girdle questionnaire (score converted to percentage 0-100%, where 0 is worst pelvic health, 100 is best pelvic health). **EQ-5D-5L**: describes quality of life through scores in 5 dimensions all scored between 1 = no problems to 5= extreme problems. **EQ-VAS** (scale from 0-100: where 0 is worst health and 100 is best health). **ASLR** = Active Straight Leg Raise test (scale 0-5 where 0 = no difficulty raising leg, 5 = severe difficulty. Measured per leg.). **ASLR range test** = Active Straight Leg Raise Range test (measures how high a straight leg can be raised from examination table to the nearest 5 degree). **6MWT** = 6 minute walking test (measures the distance in meters walked in 6 minutes in one round). **TUG** = Timed Up and Go (Measures the time it takes a patient to sit in chair, raise, walk 3 meters back and forth and sit back in chair. Mean time of two rounds is recorded).

#### **Table S2: Missing data**

|  | **Surgery**  **n=32** | | **Sham**  **n=31** | |
| --- | --- | --- | --- | --- |
|  | **3 months** | **6 months** | **3 months** | **6 months** |
| NRS operated SIJ | 1* | 0 | 1* | 0 |
| NRS global pain | 1* | 0 | 1* | 0 |
| NRS radiating pain | 1* | 0 | 1* | 0 |
| ODI | 1* | 0 | 1* | 0 |
| PGQ | 1* | 0 | 1* | 0 |
| EQ5D | 1* | 1* | 1* | 0 |
| 6MWT | 5** | 3*** | 4^α^ | 1^β^ |
| TUG | 3^ϕ^ | 2^κ^ | 4^μ^ | 1^π^ |

**Footnotes Table S3:**

*Missing due to COVID pandemic, not able to reach patients.

** Of these 5 missing: 3 are due to COVID preventing patients from coming to the hospital, 2 did not manage to complete 6MWT due to pain.

***Of these 3 missing: 1 is true missing, 1 did not complete due to pain, 1 is due to COVID preventing attendance at hospital.

^α^ Of these 4 missing: all due to COVID pandemic preventing attendance at hospital.

^β^ Can not complete 6MWT due to pain.

^ϕ^All missing due to COVID pandemic preventing attendance at hospital

^κ^ 1 missing, and 1 missing due to COVID pandemic preventing attendance at hospital

^μ^ All missing due to COVID pandemic preventing attendance at hospital

^π^ can not complete TUG due to intense pain.

#### **Table S3: Outcomes presented as mean (95% confidence interval or median, interquartile range). P-values from Students t-test or Mann-Whitney U-test.**

|  | **Baseline** | | **3 months** | | | **6 months** | | |
| --- | --- | --- | --- | --- | --- | --- | --- | --- |
|  | Surgery | Sham | Surgery | Sham | Surgery vs Sham | Surgery | Sham | Surgery vs  Sham |
|  | (Mean, 95% CI) | | (Mean, 95% CI) | | (Mean diff,  95% CI, p-value) | (Mean, 95% CI) | | (Mean diff,  95% CI, p-value) |
| **NRS operated SIJ** | **7*·*6**  (7·2 – 8·0) | **7*·*7**  (7·2 – 8·2) | **5*·*3**  (4·4 – 6·3) | **5*·*8**  (4·8 – 6·8) | **-0*·*4**  (-1·8 – 0·9)  *p=0*·*52** | **5*·*0**  (3·9 – 6·0) | **6*·*0**  (5·0 – 7·0) | **-1*·*0** (-2·4 – 0·4)  *p=0*·*14* |
| **NRS global pelvic pain** | **6*·*9**  (6·4-7·5) | **7**·**1**  (6·4 –7·8) | **5*·*7**  (4·9 – 6·4) | **6*·*1**  (5·2 -7·1) | **-0*·*4**  (-1·6 – 0·8)  *p= 0*·*9** | **5*·*8**  (5·1 – 6·6) | **6*·*3**  (5·6 – 7·1) | **-0*·*5** (-1·5 -0·5)  *p=0*·*34* |
| **NRS radiating pain** | **5*·*7**  (5·0 - 6·5) | **5*·*.8**  (4·9 – 6·7) | **4*·*4**  (3·4 – 5·4) | **4*·*6**  (3·5 – 5·6) | **-0*·*2**  (-1·5 – 1·2)  *p=0*·*80* | **4*·*4**  (3·4 - 5·4) | **4*·*8**  (3·8 – 5·9) | ***-*0*·*4** (-1·8 – 1·0)  *p=0*·*34* |
| **ODI (Sum)** | **51*·*2**  (45·9 -56·4) | **52*·*5**  (47·4 – 57·6) | **45*·*6** *  (39·1 -52·0) | **46*·*6***  (40·4 – 52·8) | **-1*·*0***  (-9·7-7·8)  *p=0*·*83* | **47**  (41 – 53) | **51**  (45 - 56) | **-4** (-12 – +4)  p=0·37 |
| **PGQ (%)** | **70*·*0**  (64·3-75·8) | **74*·*4**  (71·2 – 77·6) | **63*·*0** *  (55·5-70·6) | **66*·*3***  (59·7 - 72·9) | **-3*·*3***  (-13·1 – 6·6) *p=0.51* | **63**  (56 - 69) | **69**  (64 – 75) | **- 7** (-15 – +2)  *p=0*·*12* |
| **EQ-5D-5L index** | **0*·*63**  (0·57 – 0·68) | **0*·*61**  (0·56-0·65) | **0*·*67**  (0·61-0·73) | **0*·*67**  (0·61-0·74) | **-0*·*03**  (-0·09 – 0·08) *p=0*·*94* | **0*·*65*****  (0·59-0·72) | **0*·*65*****  (0·60-0·69) | **0*·*01*****  (-0·07 – +0·08)  *p=0*·*88* |
| **EQ-5D VAS** | **38*·*0**  (31.0 – 45.1) | **38*·*4**  (32.3 – 44.7) | **49*·*6** ***  (41.7 – 57.5) | **46*·*7*****  (37.8 – 55.5) | **2*·*9** (-8.7 – 14.5) *p=0.62* | **40*****  (32 – 47) | **43*****  (36 – 51) | **-4*****  (-14 – +6)  *p=0*·*45* |
| **6MWT** | **389**  (331 - 447) | **375**  (326-424) | **414**  (345- 483) | **442**  (401 – 484) | **29** (-107 - 50)  *p=0.47* | **455*******  (376 – 533) | **431*******  (385 – 476) | **24*******  (-64 – +112)  *p=0*·*58* |
| **TUG**** | **10*·*4**  (7·4 -13·6) | **10*·*9**  (9·3 –14·7) | **8*·*6**  (6·5 – 13·5) | **9*·*1**  (7·7 –12·0) | *p=0*·*87*** | **7*·*8**  (6·6 –11·6) | **9*·*4**  (8·0–13·0) | ***p=0·09***** |

**Footnotes Table S4:**

*n=31 in surgical group and n=30 i sham group at 3 months; **Values for median and interquartile range as not normally distributed, p-values from Mann-Whitney U-test.; *** n=28 in sham and 30 in surgical group.; **** n=29 in sham and 31 in surgical group.; ***** N=30 in sham group and 30 in surgery group

**Outcome scales Table S4:** **NRS** = Numeric Rating Scale (measured on scale 0-10 where 0 is no pain and 10 is worst imaginable pain). Minimally important difference (MID) = 2 NRS points. **ODI**  = Oswestry Disability Index (scale 0-100, where 0 is normal function,100 is bedridden). MID = 15% change . **PGQ** = Pelvic girdle questionnaire (score converted to percentage 0-100%, where 0 is worst pelvic health, 100 is best pelvic health). MID = 25 points . **EQ-5D-5L** : describes the societal view on health with index from approx. 0 (= worst health) to approx.. 1 (= best health). MID = 0.03 points . **EQ-VAS** : scale from 0-100 (where 0 is worst health and 100 is best health). MID = 10.5 points .  **6MWT** = 6 minute walking test (measures the distance in meters walked in 6 minutes in one round). MID = + 60 metres . **TUG** = Timed Up and Go (Measures the time it takes a patient to sit in chair, raise, walk 3 meters back and forth and sit back in chair. Mean time of two rounds is recorded). MID = test time change of 2.5 seconds .

|  | Baseline | | 3 months | | | 6 months | | |
| --- | --- | --- | --- | --- | --- | --- | --- | --- |
|  | Surgery | Sham | Surgery | Sham | Surgery vs Sham | Surgery | Sham | Sugrery versus Sham |
|  | Mean (95% CI) | | Mean (95% CI) | | Mean diff (95% CI)  p-value | Mean (95% CI) | | Mean diff  (95% CI)  p-value |
| NRS operated SIJ | **7·6**  (7·2 – 8·0) | **7·7**  (7·2 – 8·2) | **5·4***  (4·5 - 6·3) | **5·6***  (4·7 - 6·5) | **-0·2**  (-1·5 - 1·0)  p=0·73 | **5·0**  (4·0 – 5·9) | **6·0**  (5·1 – 6·8) | **-1·0**  (-2·2 – +0·3)  p=0·13 |
| NRS global pelvic pain | **6·9**  (6·4-7·5) | **7·1**  (6·4 –7·8) | **5·8***  (5·1 – 6·4) | **6·1***  (5·4 – 6·8) | **-0·3***  (-1·2 – 0·6)  p=0·50 | **5·9**  (5·2 – 6·5) | **6·3**  (5·6 – 6·9) | **- 0·5**  (-1·5 – 0·5) p=0·34 |
| NRS radiating pain | **5·7**  (5·0 - 6·5) | **5·8**  (4·9 – 6·7) | **4·5***  (3·6 – 5·3) | **4·6***  (3·7 – 5·5) | **-0·1***  (-1·4 – 1·1)  p=0·83 | **4·4**  (3·5 – 5·3) | **4·8**  (3·9 – 5·7) | -**0·4**  (-1·3 – +0·6)  p=0·42 |
| ODI(sum) | **51**  (46 -56) | **53**  (47 – 58) | **46***  (42·2-51·0) | **47***  (41·0 – 52·5) | **-0·5***  (-9 – 8)  p=0·90 | **47**  (43 – 52) | **50**  (46 – 54) | -**2·6**  (-9 – +4)  p=0·41 |
| PGQ (%) | **70**  (64 - 76) | **74**  (71 – 78) | **63·4***  (57·2 – 69·6) | **66·6***  (60·3 – 72·9) | **-3·2***  (-12·1 – 5·6)  p=0·47 | **64**  (59 – 69) | **68**  (62 – 73) | -**4**  (-12 – +4)  p=0·33 |
| EQ-5D-5L index (range) | **0·63**  (0·57 – 0·68) | **0·61**  (0·56-0·65) | **0·66**  (0·62 – 0·70) | **0·68**  (0·64 – 0·72) | **-0·02**  (-0·07 – 0·05)  P=0·77 | **0·65**  (0.60 – 0.69) | **0·66**  (0.61 – 0.7) | **-6·8**  (-15·6 – +2·0)  p=0·13 |
| EQ-5D VAS  (Scale) | **38**  (31 – 45) | **38**  (32 – 45) | **49·0****  (41·8-56·2) | **47·1****  (39·6 – 54·5) | **1·9****  (-8·4 – 12·3)  p=0·72 | **40*****  (34 - 47) | **43*****  (37 - 50) | **-3·2** (-13– +6)  p=0·51 |
| 6MWT (metres) | **389**  (331- 447) | **375**  (326-424) | **421******  (364 – 477) | **439******  (382 – 496) | **-18******  (-98 - 62)  p=0·65 | **448*******  (491 - 405) | **435*******  (391 - 478) | **+13·7*****  (-48 – +75)  P=0·66 |
| TUG | **10·4**  (7·4 -13·6) | **9·4**  (8·0 –13·0) | **13·2**  (11·2 – 15·2) | **11·1**  (9·1 – 13·2) | **2·0**  (-0·8 – 4·9)  p=0·82 | **10·6**  (8·6 – 12·5) | **11·7**  (9·8 – 13·7) | **-1·2*******  (--4·0 - +1·6)  p=0·41 |

#### **Table S4: Outcomes presented as mean (95% confidence interval) with p-values from a linear mixed model (with a subject-specific random intercept and with the outcome variable at baseline, time, intervention groups and the interaction between time and intervention groups as fixed effects).**

**Footnotes table S5:**

*N=30 in sham group and 31 in surgery group; ** N= 28 in sham group and 30 in surgery; *** N=29 in sham group and 31 in surgery group; **** N=27 in sham group and 27 in surgery group; ***** N=30 in sham group and 30 in surgery group

**Outcome scales table S5:** **NRS** = Numeric Rating Scale (measured on scale 0-10 where 0 is no pain and 10 is worst imaginable pain). Minimally important difference (MID) = 2 NRS points. **ODI**  = Oswestry Disability Index (scale 0-100, where 0 is normal function,100 is bedridden). MID = 15% change . **PGQ** = Pelvic girdle questionnaire (score converted to percentage 0-100%, where 0 is worst pelvic health, 100 is best pelvic health). MID = 25 points . **EQ-5D-5L** : describes the societal view on health with index from approx. 0 (= worst health) to approx.. 1 (= best health). MID = 0.03 points . **EQ-VAS** : scale from 0-100 (where 0 is worst health and 100 is best health). MID = 10.5 points .  **6MWT** = 6 minute walking test (measures the distance in meters walked in 6 minutes in one round). MID = + 60 metres . **TUG** = Timed Up and Go (Measures the time it takes a patient to sit in chair, raise, walk 3 meters back and forth and sit back in chair. Mean time of two rounds is recorded). MID = test time change of 2.5 seconds .
