# Supplementary material for: The effect of minimally invasive sacroiliac joint fusion compared to sham operation: a double-blind randomized placebo-controlled trial
Source: eClinicalMedicine. 2024 Feb 1;68:102438. doi: 10.1016/j.eclinm.2024.102438 (PMC10847054; doi:10.1016/j.eclinm.2024.102438)
Supplement: Data Sharing Statement [file mmc2.docx]

# Data Sharing Statement

Raw trial data from this clinical trial is not available for data sharing due to Norwegian law regulations.

| Data sharing question: | Protocol and statistical analysis plan | Raw data from trial |
| --- | --- | --- |
| Will individual participant data be available (including data dictionairies)? | Not applicable | No |
| What data in particular will be shared? | Not applicable | Not applicable |
| What other documents will be available? | Study protocol and statistical analysis plan | Not applicable |
| When will data be available (start and end dates)? | Immediately following publication; no end date | Not applicable |
| With whom? | Study protocol and statistical analysis plan available for anyone who whishes to access these documents. | Not applicable |
| For what type of analyses? | Not applicable | Not applicable |
| By what mechanism will data be made available? | Study protocol and statistical analysis plan are available indefinitely at (link to be included). | Not applicable |
