# Supplementary material for: The effect of minimally invasive sacroiliac joint fusion compared to sham operation: a double-blind randomized placebo-controlled trial
Source: eClinicalMedicine. 2024 Feb 1;68:102438. doi: 10.1016/j.eclinm.2024.102438 (PMC10847054; doi:10.1016/j.eclinm.2024.102438)
Supplement: SIFSO Supplement Protocols and SAP SIFSO [file mmc3.pdf]

Supplement to Sacroiliac joint fusion compared to sham operation – a double blind RCT.

Author:
Randers, Engelke MD

<u>This supplement contains the following items:</u>

1. Original protocol (p.4-20), final protocol (p.21-42), Summary of changes (p.43)
2. Original statistical analysis plan (p.44), final statistical analysis plan (p.44), Summary of changes (p.45)

Table of contents:

Original protocol Version 001, date 31.august 2017 3
Table of contents *4*
Introduction *4*
Study aims *7*
Research group *7*
Subject enrollment *8*
Recruitement and feasibility *8*
Methods *9*
Study design 9
Randomization 9
Endpoints 9
Inclusion and exclusion criteria 10
Interventions 12
Study procedures 13
Study Flow Chart *15*
.... *15*
Statistics *16*
Power calculation *16*
Ethical considerations *16*
Publication plan *18*
Plan of progress *18*
References *18*
FINAL VERSION OF SIFSO PROTOCOL VERSION 3 Dated 30th June 2022 21
Introduction *23*
Patients and methods *24*
Study design 24
Patients 24
Interventions 26
Sample size 28
Outcomes 29

Study procedures 30
Data analysis plan 31
DISCUSSION *38*
Ethics, registration, funding and potential conflicts of interest *39*
Aknowledgements *39*
References: *39*
Changes between original protocol and final protocol 43
Original Statistical analysis plan dated august 2017: 44
Final Statistical analysis plan dated 30th June 2022: 44
Changes in statistical analysis plan 45

Original protocol Version 001, date 31.august 2017:

SacroIliac Joint Fusion vs Sham Operation (SIFSO) for treatment of sacroiliac joint pain A prospective double blinded randomized controlled multicenter trial

Engelke Randers, MD
Jon Dahl, MD, PhD
Thomas Johan Kibsgård, MD, PhD
Oslo University Hospital, Department of Orthopaedics, Oslo, Norway

Paul Gerdhem
Karolinska University Hospital, Stockholm, Sweden
Andreas Westberg, MD, St. Göran Hospital
St. Göran Hospital, Department of Orthopaedics, Stockholm, Sweden

Table of contents

Introduction……………………………………………………………………….4
Study aims…………………………………………………………………………...7
Research group……………………………………………………………………7
Subject enrollment…………………………………………………………………..8
Recruitement and feasibility………………………………………………………...8
Methods……………………………………………………………………………...9
Study flow chart……………………………………………………………………15
Statistics……………………………………………………………………………16
Ethical considerations……………………………………………………………...16
Publication plan……………………………………………………………………18
Plan of progress……………………………………………………………………18
References…………………………………………………………………………18

Introduction

The sacroiliac joint (SIJ) might be the source of pain for 13-30% of patients with low back pain and possibly an even greater proportion of patients suffering from “failed back surgery” (1-3). This pain can be caused by specific pathology of the joint (4), but the role of the SIJ in unspecific pelvic girdle pain disorder remains unclear. The first treatment of choice for patients with SIJ pain is non-surgical treatment (3). The treatment options include physical therapy, pain medication, SIJ steroid injections and radiofrequency ablation of the SIJ. Although conservative care is the first choice of treatment, some patients unfortunately do not respond to this treatment, or only have a transient improvement and end up with long-lasting severe pain and severely reduced physical function (3, 5-8).

Patients with severe SIJ pain are shown to have a severely reduced quality of life and high pain intensity compared with a healthy age and gender equal control group (9). In a previous study from our department the patients that were assigned for SIJ surgery scored a mean of 84 in the evening, measured by a 0-100 visual analogue pain scale (scale of 0-100, 0= no pain, 100 = worst possible pain), and reported severe disability in functional outcome questionnaires (6). The mean age of these patients were 40 years and they were all on disability leave. Severe SIJ can affect young patients, especially women with pelvic girdle pain after pregnancy, and the disease may affect their family life and working capabilities significantly. Of the last 17 patients we have treated with SIJ fusion in our department, 40% could not walk without churches and the other 60% could not walk more than 100-250 meters. Patients with SIJ pain have intolerance to physical activity and great variability in symptom intensity (6). Consequently, during the worst periods of pain intensity, the majority

of the patients have to use crutches and some also use a wheelchair in their daily life in order to be mobile. The burden of disease in SIJ patients is worse than other chronic illnesses, such as chronic obstructive pulmonary disease, coronary heart disease, asthma and mild heart failure(9). More importantly, the impact of SIJ pain, eligible for surgery, appears to be similar that of other orthopedic surgical conditions, such as hip osteoarthritis, degenerative spondylolisthesis, spinal stenosis and knee osteoarthritis(9). These are all recognized, debilitating orthopedic conditions that affect physical function, lead to use of walking aids and affects quality of life in general. This demonstrates that patients with SIJ pain eligible for surgery have a high level of disability and severe pain and therefore a poor general health and lowered quality of life compared to the reference standard.

The role of the SIJ as a pain generator has interested orthopedic surgeons for almost a century. Smith-Petersen described a method for SIJ fusion in 1921 (10), and since then, several different surgical procedures have been tried to treat patients with suspected SIJ pain. During the first decades, a large proportion of the patients had joint infections (e.g tuberculosis), and many of the first surgical techniques were developed to treat these infections. Patients with "pelvic relaxation" during pregnancy have also comprised a large proportion of the patients receiving SIJ fusion (10-14). Before the role of the herniated disc was discovered, SIJ fusion was a novel treatment for low back pain and sciatic pain. The same accounts for pelvic disruption after pregnancy. Treatments for these conditions were described in several case series between 1921 and the 1940s (10-14). After the 1940s, there were no papers in the literature until the 1970s, when new reports of SIJ fusion started to appear. Most of the papers report a great portion of successful cases, but also have a significant fraction of failures. Many orthopedic surgeons who performed SIJ fusions did however not achieve the results they wanted. Because the outcomes were unpredictable and the complication rate appeared unacceptably high, the enthusiasm diminished during the 1980-90s. In recent years, the role of the SIJ in orthopedic surgery has again gained popularity. This new wave of popularity is primarily driven by the introduction of minimal invasive surgery (MIS) (7, 8, 15).

It has been questioned whether MIS implants can improve the outcomes of SIJ fusion. Over the last decade, several minimally invasive procedures have been introduced. The use of these SIJ implants has increased despite lack of high quality documentation (16). In the last few years, several manufacturers have created MIS implants for use in SIJ fusion, but studies have been published for only three of these implants (7, 8, 17-20) . In the last two to three years several publications have been published and most of them report promising results (5, 21,

22). A majority of the papers are case series mostly published by the industry or by authors with close connections to the industry. In 2015 and 2016 two RCT's were published (7, 8, 23). In these studies the patients with SIJ fusion reported a significant decrease in pain and disability. The fused patients did much better than the conservatively treated control group. There are significant limitations to these studies, but the fact that several studies report successful outcome with low complication rate cannot be ignored.

Three orthopedic centers in Scandinavia operate patients with SIJ pain. Oslo University Hospital (Norway), Ängelholm Hospital (Sweden) and St. Göran Hospital (Sweden). In Sweden approximately 100-150 patients are operated each year and in Norway 10-20 patients undergo surgery. Although only a small number of patients are operated each year, it is clear that the patients with SIJ pain have a high degree of disability and pain (7, 8). These patients experience a large burden of their disease and the pain influences these patients life to a large degree. According to the Ängelholm study by Sturesson et al. (2016) the results after SIJ fusion are comparable to other spinal procedures. Westberg (St.Göran Hospital) presented his data on 50 consecutive SIJ fusion at the "World Congress of Low Back and Pelvic pain" in Singapore (2016). He presented comparable results to Sturesson et al (2016). Judging from these studies it seems that if the patients are selected properly, one may treat patients with severe SIJ pain with surgical fusion of the SIJ.

Results from two RCT's have been published (7, 8). In both studies conservative care was compared to the new MIS procedure. The primary endpoint in both studies was pain reduction after six months. The group that received conservative care had only a minor positive change compared to the SIJ fusion group. After six months the majority of patients in the control group crossed over to the SIJ fusion group and experienced the same benefit from surgery. There are some limitations with the study design in these studies. The patients in the conservative group were told that if they did not have any effect of conservative care after six months, they would be allowed to cross over to the fusion group. One can therefore question the motivation to get well in the conservative group since most of the patients had already tried conservative care with no effect on pain and disability. Some of the experienced effect of the new MIS treatment may be explained by placebo.

The placebo effect after surgery has been shown to be an important factor in short-term efficacy. This has particularly been shown in treatment of chronic pain where the psychological component is believed to be an important factor (24). For example, in a study using sham surgery as placebo, 43% of the patients in the placebo group had pain relief after sham lumbar discectomies (25). Other similar sham studies have also reported to have a

significant effect on clinical outcome (26). There are several factors in both patients and examiners that contribute to the placebo effect. Patients with long-lasting diseases seem to be poorer placebo responders, but on the other hand placebo tend to work better in patients expecting to have changes in sensation of pain (26). Although surgical treatment of SIJ has been shown to have effect superior to conservative care, there is still a question if these effects can be explained by placebo.

Study aims

The aim of the study is to examine if there is a difference in reduction in SIJ pain between patients treated with minimal invasive SIJ fusion compared to a sham operation without fusion of the sacroiliac joint. Our null-hypothesis is that there is no difference in SIJ pain between the groups after six months.

Research group

This study will be conducted in two orthopaedic centers; Oslo University Hospital, Oslo, Norway and Karolinska University Hospital, Stockholm, Sweden. The main academic center will be Oslo University hospital and the project will be led by the principle investigator, Thomas Johan Kibsgård.

PhD candidate:
Engelke Randers, MD
Resident orthopedic surgeon, Department of Orthopedics, Oslo University Hospital, Oslo, Norway.

Principle investigator and main supervisor:
Thomas Kibsgård, MD, PhD,
Head of Department of spinal deformities, Department of Orthopedics, Oslo University Hospital, Oslo, Norway
Associate professor, University of Oslo, Oslo, Norway

Supervisors:
Jon Dahl, MD, PhD
Consultant orthopaedic Surgeon, Department of Orthopedics, Oslo University Hospital, Oslo, Norway
Britt Stuge, PT

Associate professor, University of Oslo, Norway

Collaborations outside Norway:
A collaboration with Karolinska University Hospital and St. Göran Hospital, Stockholm, Sweden has been made and the project will be led by Paul Gerdhem, Professor at Karolinska University Hospital, and Andreas Westberg, MD, St. Göran Hospial.

Subject enrollment

Each participant will be assigned a unique and anonymous study number at the time of inclusion. This number will be retained throughout the study. The study site will keep a log with patient name and study number. All study records will be evaluated and tracked using only the unique study number. The study number and informed consent will be stored and locked until the end of the study.

Recruitement and feasibility

The orthopaedic department at Oslo University Hospital (OUS) is currently the only center in Norway that performs SIJ fusion. Approximately 40 patients are evaluated in the outpatient clinic at OUS for SIJ pain per year and approximately 10-15 patients are operated with SIJ-fusion each year. However, this is a group of patients that have been overlooked for many years in Norway and with wider recognition of the condition we expect an increase in the number of referred patients for evaluation, which again will lead to an increase in potential study candidates.
Still, due to a suspected limited number of study subjects in Norway, we will have a cooperating study center in Sweden. This will give us a larger population to recruit patients from and make us able to perform the study within a shorter time-frame. With an estimated sample-size of 60 patients we assume that all study candidates can be recruited in Norway within two to three years. However, involving another centre will enable us to complete the recruiting period in approximately one year. All recruitment will happen after oral and written study information is given, and with written consent from the patient.

Patients with SIJ pain are predominantly female and are patients with chronic pain motivated for any treatment that can lead to pain reduction and better function in daily life. They have often tried multiple conservative treatments with poor result. They are therefore more likely to be motivated for repeated evaluations/examinations and surgical treatment if there is

indication for this. It is also shown that females show greater compliance than men with regard to follow-up visits, examinations, and completing and returning research forms. The patients will be in close contact with the study-group at their contact hospital throughout their follow-up time.

Methods

Study design

This two-arm trial is a multicenter placebo-controlled double blinded trial (patient and outcome assessor blinded) in which patients are randomized to active surgery or sham surgery (placebo).

Randomization

Each patient will be evaluated and included in the outpatient clinic where they receive a baseline assessment. After the baseline assessment is complete, the study subject will receive a date for surgery. When the patient is in the operating room under general anesthesia he/she will be randomly assigned to either iFuse or sham operation. Randomization will be blocked by study center with random block sizes of 4 or 6. Subjects will be assigned in a 1:1 iFuse:sham ratio. Randomization will be performed using a study-specific website (VieDoc from OUS). The website displays the randomization assignment and simultaneously sends an email to the supervisor. Study subject, researcher/investigator/coordinator are blinded. Only the surgeon knows the result of the randomization until trial primary endpoint at six months and he will not be part of the follow up of the patients.

Endpoints

Primary Endpoint

The primary endpoint is change in Numeric Rating Scale (NRS) pain-score on the operated side at six months postoperatively (27).

Secondary Endpoints

The study's secondary endpoints are:

- Change from baseline in Numeric Rating Scale (NRS) pain-score on the operated side at 3, 6, 12 and 24 months post-op.
- Change from baseline in global NRS at 3, 6, 12 and 24 months
- Change from baseline in non-operated side NRS at 3,6,12 and 24 months
- Change from baseline in leg pain NRS at 3, 6, 12 and 24 months

- Change in disability due to pelvic pain measured by Oswestry Disability Index (ODI) at 3, 6, 12 and 24 months (28).
- Change in disability due to pelvic pain measured by Pelvic Girdle Questionnaire at 3, 6, 12 and 24 months (29).
- Change in quality of life (EQ-5D) at 3, 6, 12 and 24 months
- Device breakage, loosening or migration at 12 months (CT)

In addition to the predefined endpoints there will be collected information about adverse events and changes in objective clinical tests, ambulatory status and work status.

The NRS on the operated side was chosen as our primary endpoint because of multiple factors. First of all, NRS has been used in the two previous RCTs where fusion of the SI-joint has been compared to conservative treatment and also in many other studies on clinical outcome after SIJ fusions(5, 7, 8). This makes it easy to compare our result to existing literature.
Secondly, it has been reported that the NRS responsiveness is better than the commonly used visual analogue pain scale when these types of pain scales are used in chronic patients (27).

We also find that most of the patients suffering for SI-joint pain are capable of pinpointing their pain to either side (the ability to this is also an inclusion criteria), and often describe their pain as a knifelike pain at the posterior superior iliac spine (PSIS/SIPS). They are therefore capable of differentiating the pain from either SI-joint and give an NRS for each side as well as a global NRS. In our local register we see that the patients with bilateral pain score reduction in NRS on the operated side but fail to report decrease in global disability and pain due to pain in the contra lateral SIJ. With the use of NRS on the operated side we can include patients with bilateral condition.
We therefore find this to be a good primary endpoint for our study as we wish to find whether there is pain reduction in the operated SI-joint.

Inclusion and exclusion criteria
Patients will be eligible for the study if they meet all of the inclusion and none of the exclusion criteria listed in table below.

Inclusion Criteria

1. Age 21-70 at time of screening
2. Patient with suspected SIJ pain for >6 months or >18 months for pregnancy induced pelvic girdle pain.
3. Diagnosis of the SI joint as the primary pain generator based on ALL of the following:
   A. Patient has pain at or close to the posterior superior iliac spine (PSIS) with possible radiation into buttocks, posterior thigh or groin and can point with a single finger to the location of pain (Fortin Finger Test)
   B. Patient has at least 3 of 6 physical examination maneuvers specific for SI joint pain
      1. Compression
      2. Posterior Pelvic Pain Provocation test – P4 (Thigh Thrust)
      3. Palpation of the long dorsal ligament
      4. Patrick's test (Faber)
      5. Leg Raise (ASLR)
      6. Geanslens test
   C. Patient has improvement in lower back pain NRS of at least 50% of the pre injection NRS score after fluoroscopic controlled injection of local anesthetic into affected SI joint (including previous documented test <6 months ago)
4. Baseline Oswestry Disability Index (ODI) score of at least 30%
5. Baseline lower back pain score of at least 5 on 0-10 point NRS
6. Patient should have tried adequate forms of conservative treatment with little or no response.
7. Patient has signed study-specific informed consent
8. Patient has the necessary mental capacity to participate and is physically able to comply with study protocol requirements.
9. Patients with unilateral or bilateral pain can be included in the study if they clearly can differentiate between the two sides. It is the most painful side that will be included and randomized to SIJ fusion or sham surgery in the study.

Exclusion Criteria

1. Severe low back pain due to other causes, such as lumbar disc degeneration, lumbar disc herniation, lumbar spondylolisthesis, lumbar spinal stenosis, lumbar facet degeneration, and lumbar vertebral body fracture.
2. Sacroiliac pathology caused by auto-immune disease (e.g. ankylosing spondylitis) and/or neoplasia (e.g. benign or malignant tumor) and/or crystal arthropathy

3. History of recent (<1 year) fracture of the pelvis with documented malunion, non-union of sacrum or ilium or any type of internal fixation of the pelvic ring.
4. Spine surgery during the past 12 months.
5. Previously diagnosed or suspected osteoporosis (defined as prior T-score <-2.5 or history of osteoporotic fracture)
6. Documented osteomalacia or other metabolic bone disease
7. Any condition or anatomy that makes treatment with the iFuse Implant System infeasible
8. Patients with prior successful fusion to the contra lateral side are exluded from the study.

Interventions

All patients will receive the same pre- and postoperative assesment (i.e. blood samples, general anesthesia, draping, wound care and pain medication). They will not be randomized before they are under anesthesia and in prone position on the operating table. The operations will be done by two surgeons and the theater will be closed to all other personnel and the windows to the theater will be covered. Two scrub nurses and one anesthesia nurse will be in the room during the whole procedure.

After the procedure is completed the patients will be treated as if he/she has undergone the actual procedure with the post- operative care that implies.

The post-operative follow up during the hospital stay will be done by blinded health staff.

Surgical Procedure

Subjects randomized to treatment with iFuse (intervention group).

The procedure starts with an approximately 5cm long skin incision over the posterolateral aspect of the pelvis. A guide-pin is inserted over the iliosacral joint at the desired entry-point, verified by fluoroscopy. The surgeon drills and broaches over the pin, and the iFuse implant is inserted. This is repeated for a total of three implants. The wound is closed with non-resorbable suture. An injection of the SIJ with Marcain / Lederspan is performed under guidance of fluoroscopy after closure.

Details of the procedure are available in the Surgical Technique Manual (https://si-bone.com/patients/ifuse-implant-system/video-watch-the-ifuse-surgery/). The investigator and/or coordinator will document key aspects of the surgical procedure in a study-specific case report form (CRF).

Sham operation

The patient randomized to a sham operation (control group).

The sham operation will consist of the surgeon making the same skin incision as for an iFuse procedure, although nothing more and then closing the wound.

The patients undergoing a sham operation will be under general anesthesia for a random time of 20-40 minutes in order to keep the two procedures as similar as possible.

An injection of the SIJ with Marcain / Lederspan is performed under guidance of fluoroscopy after closure.

Study procedures

Screening

Patients will be screened for study eligibility criteria during evaluation in the outpatient clinic.

A signed study-specific informed consent form (ICF) must be obtained from each potential participant before performing any test that goes beyond standard clinical examination.

Physical Examination Maneuvers

Standard physical examination maneuvers are used to diagnose pain as emanating from the SI joint. To be eligible for the study subjects must have at least 3 of the 6 following physical tests positive:

1. Compression or distraction
2. Posterior Pelvic Pain Provocation test – P4 (Thigh Thrust)
3. Palpation of the long dorsal ligament
4. Patrick's test (Faber)
5. ASLR
6. Geanslens

A positive test is one that reproduces the subject's pain. Physical examination findings will be recorded on baseline CRFs.

Radiology

All patients will undergo CT preoperatively and the fused patients will, after the blinding is revealed, undergo pelvic CT after six months. No postoperative radiological control will be performed; the reason for this is not to disclose which group the patient was randomized to. Correct placement of the iFuse implant will be confirmed with fluoroscopy in the operating room. If there is a medical reason to violate this (e.g. neurological deficiency) the patient will be excluded from the study.

Postoperative Management

All study subjects will receive standard in-hospital postoperative care including pain medication and anti-thrombotic medication. The patients can normally leave the hospital on

the first postoperative day. They will be instructed to partial weight-bearing on crutches for 4 weeks, and an additional two weeks on crutches with weight bearing up to the pain limit.

Study Flow Chart

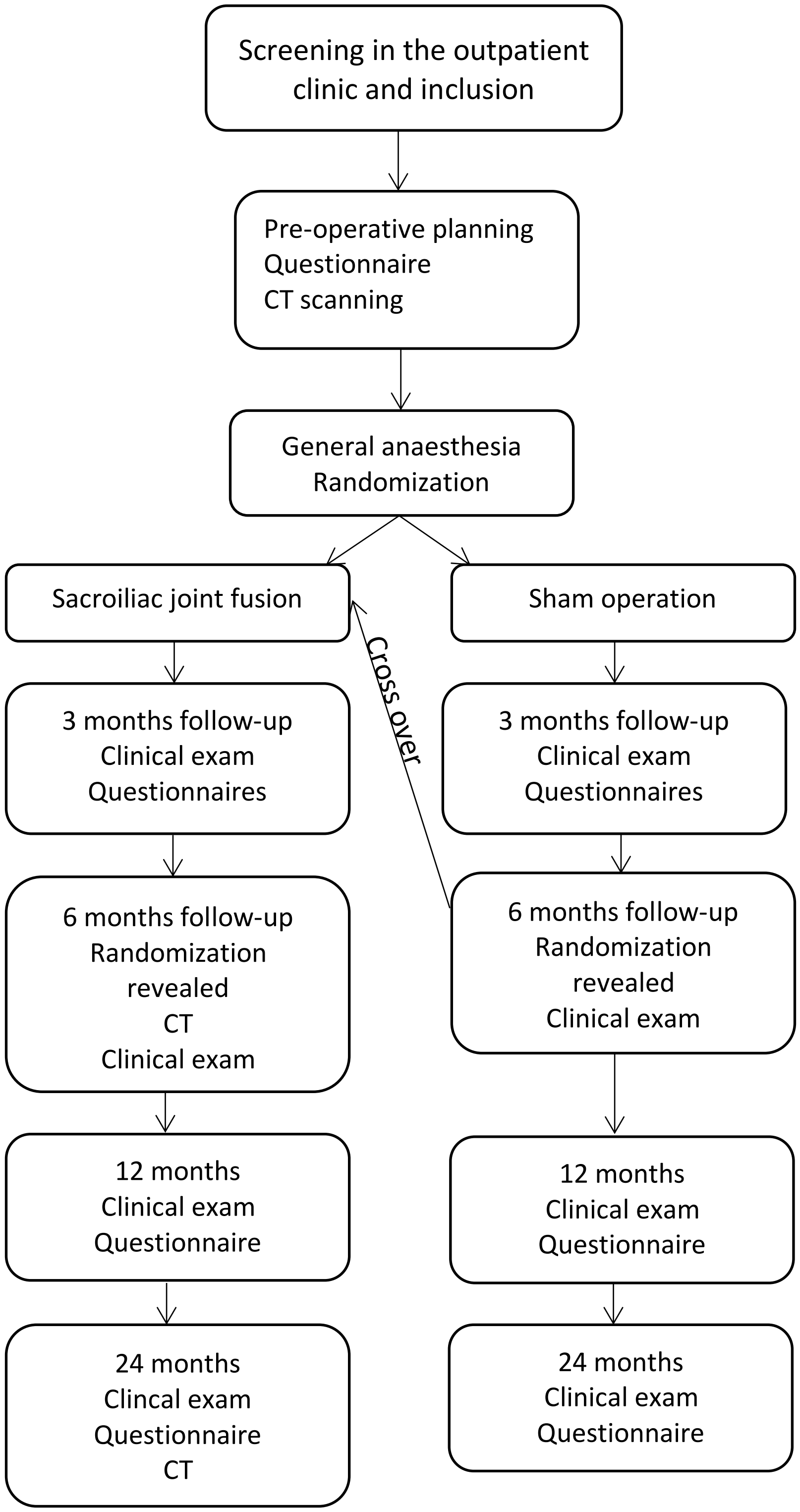

Statistics

The outcomes (both primary and secondary outcomes) will be analyzed with parametrical statistical methods.

Data from the different groups will be compared based on the 'intention to treat' principle. An intention to treat (ITT) analysis means that all patients, regardless of loss of follow-up or drop-out, remain in the analysis of the group to which they were randomized (30). Multiple imputation of missing data will be used in the ITT analysis. A sensitivity analysis will be performed comparing the ITT data against a per-protocol data exclusively from patients who complied with the study protocol. Continuous and discrete parameters will be measured using parametric or non-parametric tests (depending on skewness) for group comparisons. Categorical parameters will be compared by the Chi-square test.

We will perform blinded statistical analyses (31), in which the individual performing the analyses is unaware of group belonging. The code for group belonging will not be broken until the analyses and interpretations have been performed.

Power calculation

The power calculation was done using the primary endpoint of 2 points decrease in NRS on the operated side six months postoperatively. Previous reports have shown that 2 points reduction in NRS pain scale probably represents a clinical important difference (32, 33). The standard deviation was set to 2,5 points since the SD has been reported in previous studies to vary between 1,0-2,9 at 6 months follow up after SIJ fusion (7, 12, 22, 34). The average mean preoperative NRS pain score in previous studies has been reported to be 7,0-8,5 (7, 12, 22, 34). The probability of type 1 error (alpha) was set to 0.05. We assume that the fusion group will have at least 3 points decrease in NRS. The two available RCT's report decrease in global NRS of respectively 4,3 and 5,3. The placebo group was estimated to have 1 point decrease. The same two studies show 0,5-1,3 points decrease in NRS in patients treated with conservative care. Based on these assumptions we calculate 25 patients in each group with 80% power using an online sample size calculator (www.clincalc.com). Due to a possible dropout of 20% we calculate the total number of participants to be 60 patients.

Ethical considerations

The study needs to be approved by the regional ethical committee for research (REK Helse Sør-Øst).

We think it is important to conduct this study to evaluate the effect of SIJ fusion. Most surgeries today are performed on an elective basis to improve quality of life and not to save lives. Fusion of the SIJ is not a lifesaving procedure, but is thought to improve quality of life for at patient group with severe pain and reduced quality of life. Although this study require 50% of the study subjects to go through a sham procedure with the potential risk this entails (e.g. general anesthesia, infection and bleeding) , we deem the risks so low that it may justify the study design. In a register study concerning almost 1,4 million otherwise healthy individuals (ASA 1 or 2) undergoing elective general anesthesia, the risk of an anesthesia related death was 7.3 per 1 million cases (35).

Another ethical aspect that we wish to highlight is the fact that this new minimal invasive procedure is being performed on thousands of patients worldwide even though there is very limited documentation on its effect. Considering this, we find it important to complete this study to evaluate whether the so-far documented pain-reduction with MIS SIJ fusion is real, or affected by a placebo component. A result showing no difference between the groups (= null hypothesis) will be of importance to the general SIJ patient population and have positive socioeconomic implications. Orthopedic surgeons will have reason not to expose patients for a procedure that incurs significant risk and has no benefit. In addition, financial resources can be saved if the procedure is shown to be ineffective. A result showing SIJ fusion to reduce pain in greater extent than a sham surgery (falsification of the null hypothesis), will support the growing practice of SIJ fusion. There are unfortunately several well accepted orthopedic procedures, which have been performed in large numbers over the years, that have recently been stopped because of well performed sham studies (36-38).

One additional treatment is applied to the sham group. They have to use churches for 4-6 weeks. To our knowledge there is no documentation that this short period of time with churches can harm the patients in the sham group. As mentioned in the introduction, a majority of these patients use churches already from time to time, and a short period of time with churches could probably help them more than harm them.

The major ethical aspect of this sham surgery would be exposing patients for general anesthesia for no reason other than a sham operation. However, we find that this study is of such an importance that this outweighs the minimal potential for complications during or after general anesthesia.

Publication plan

We expect the study to give answer to whether fusion of the SI joint has greater effect on pain reduction than sham surgery (Placebo) at 6 months. The results will be published in a peer-reviewed international journal. We will also publish results at 12 and 24 months in a second follow-up article.

Information about the study and its results will be implemented through lectures at different orthopedic fora and conventions.

Plan of progress

Start inclusion in 2017. Inclusion of patients will be from 2017 through 2018. Follow-up will be performed continuously after protocol. We expect the last six month follow-up to be completed during 2019. After last follow-up we will perform statistical analyses of data and results, and report these in an article as soon after last follow-up as possible.

References

1. DePalma MJ, Ketchum JM, Saullo TR. Etiology of chronic low back pain in patients having undergone lumbar fusion. Pain Med. 2011;12(5):732-9.
2. Simopoulos TT, Manchikanti L, Singh V, Gupta S, Hameed H, Diwan S, et al. A systematic evaluation of prevalence and diagnostic accuracy of sacroiliac joint interventions. Pain Physician. 2012;15(3):E305-E44.
3. Vleeming A, Albert HB, Ostgaard HC, Sturesson B, Stuge B. European guidelines for the diagnosis and treatment of pelvic girdle pain. EurSpine J. 2008;17(6):794-819.
4. Bellamy N, Park W, Rooney PJ. What do we know about the sacroiliac joint? SeminArthritis Rheum. 1983;12(3):282-313.
5. Duhon BS, Cher DJ, Wine KD, Lockstadt H, Kovalsky D, Soo CL. Safety and 6-month effectiveness of minimally invasive sacroiliac joint fusion: a prospective study. Med Devices (Auckl). 2013;6:219-29.
6. Kibsgård TJ, Røise O, Stuge B. Pelvic joint fusion in patients with severe pelvic girdle pain–a prospective single-subject research design study. BMC musculoskeletal disorders. 2014;15(1):85.
7. Polly DW, Swofford J, Whang PG, Frank CJ, Glaser JA, Limoni RP, et al. Two-Year Outcomes from a Randomized Controlled Trial of Minimally Invasive Sacroiliac Joint Fusion vs. Non-Surgical Management for Sacroiliac Joint Dysfunction. International Journal of Spine Surgery. 2016;10.
8. Sturesson B, Dengler J, Kools D, Pflugmacher R, Prestamburgo D. Sacroiliac minimal invasive fusion compared to physical therapy: six-month outcome from a multicentre randomised controlled trial. The Spine Journal. 2016;16(4):S73-S4.
9. Cher D, Polly D, Berven S. Sacroiliac joint pain: burden of disease. Medical Devices (Auckland, NZ). 2014;7:73.
10. Smith-Petersen MN, Rogers WA. End-Result Study of Arthrodesis of the Sacro-Iliac Joint for ArthritisGÇöTraumatic and Non-Traumatic. The Journal of Bone & Joint Surgery. 1926;8(1):118-36.
11. Hagen R. Pelvic girdle relaxation from an orthopaedic point of view. Acta OrthopScand.

1974;45(4):550-63.

12. Kibsgard TJ, Roise O, Sudmann E, Stuge B. Pelvic joint fusions in patients with chronic pelvic girdle pain: a 23-year follow-up. EurSpine J. 2013;22(4):871-7.

13. Miltner LJ, Lowendorf CS. Low Back Pain: A Study of 525 Cases of Sacro-Iliac and Sacrolumbar Sprain. The Journal of Bone & Joint Surgery. 1931;13(1):16-28.

14. Mitchell JI. Surgical treatment of affections of the lumbo-sacral and sacroiliac joints. Surgery. 1938;4:33-43.

15. Harrison DE, Harrison DD, Troyanovich SJ. The sacroiliac joint: a review of anatomy and biomechanics with clinical implications. JManipulative Physiol Ther. 1997;20(9):607-17.

16. Miller LE, Reckling WC, Block JE. Analysis of postmarket complaints database for the iFuse SI Joint Fusion System(R): a minimally invasive treatment for degenerative sacroiliitis and sacroiliac joint disruption. Med Devices (Auckl). 2013;6:77-84.

17. Al-Khayer A, Hegarty J, Hahn D, Grevitt MP. Percutaneous sacroiliac joint arthrodesis: a novel technique. JSpinal DisordTech. 2008;21(5):359-63.

18. Endres S, Ludwig E. Outcome of distraction interference arthrodesis of the sacroiliac joint for sacroiliac arthritis. Indian J Orthop. 2013;47(5):437-42.

19. Khurana A, Guha AR, Mohanty K, Ahuja S. Percutaneous fusion of the sacroiliac joint with hollow modular anchorage screws: clinical and radiological outcome. JBone Joint SurgBr. 2009;91(5):627-31.

20. Mason LW, Chopra I, Mohanty K. The percutaneous stabilisation of the sacroiliac joint with hollow modular anchorage screws: a prospective outcome study. EurSpine J. 2013;22(10):2325-31.

21. Cummings J, Capobianco RA. Minimally invasive sacroiliac joint fusion: one-year outcomes in 18 patients. AnnSurg InnovRes. 2013;7(1):12.

22. Sachs D, Capobianco R. Minimally invasive sacroiliac joint fusion: one-year outcomes in 40 patients. AdvOrthop. 2013;2013:536128.

23. Polly DW, Cher D, Wine K, Whang P, Frank C, Lockstadt H, et al. Randomized Controlled Trial of Minimally Invasive Sacroiliac Joint Fusion Using Triangular Titanium Implants vs. NonSurgical Management for Sacroiliac Joint Dysfunction. Global Spine Journal. 2016;6(S 01):GP113.

24. Turner JA, Deyo RA, Loeser JD, Von KM, Fordyce WE. The importance of placebo effects in pain treatment and research. JAMA. 1994;271(20):1609-14.

25. Spangfort EV. The lumbar disc herniation. A computer-aided analysis of 2,504 operations. Acta OrthopScandSuppl. 1972;142:1-95.

26. Vandana R, Tushar R. Placebos: current status. Indian journal of pharmacology. 2001;33(6):396-409.

27. Grotle M, Brox JI, Vollestad NK. Concurrent comparison of responsiveness in pain and functional status measurements used for patients with low back pain. Spine (Phila Pa 1976). 2004;29(21):E492-E501.

28. Grotle M, Brox JI, Vollestad NK. Cross-cultural adaptation of the Norwegian versions of the Roland-Morris Disability Questionnaire and the Oswestry Disability Index. JRehabilMed. 2003;35(5):241-7.

29. Stuge B, Garratt A, Krogstad JH, Grotle M. The pelvic girdle questionnaire: a condition-specific instrument for assessing activity limitations and symptoms in people with pelvic girdle pain. PhysTher. 2011;91(7):1096-108.

30. Hollis S, Campbell F. What is meant by intention to treat analysis? Survey of published randomised controlled trials. BMJ. 1999;319(7211):670-4.

31. MacCoun R, Perlmutter S. Blind analysis: Hide results to seek the truth. Nature. 2015;526(7572):187-9.

32. Hagg O, Fritzell P, Nordwall A. The clinical importance of changes in outcome scores after treatment for chronic low back pain. EurSpine J. 2003;12(1):12-20.

33. Farrar JT, Young JP, LaMoreaux L, Werth JL, Poole RM. Clinical importance of changes in chronic pain intensity measured on an 11-point numerical pain rating scale. Pain. 2001;94(2):149-58.

34. Duhon BS, Bitan F, Lockstadt H, Kovalsky D, Cher D, Hillen T, et al. Triangular titanium implants for minimally invasive sacroiliac joint fusion: 2-year follow-up from a prospective multicenter trial. International journal of spine surgery. 2016;10.

35. Schiff J, Welker A, Fohr B, Henn-Beilharz A, Bothner U, Van Aken H, et al. Major incidents and complications in otherwise healthy patients undergoing elective procedures: results based on 1.37 million anaesthetic procedures. British journal of anaesthesia. 2014;113(1):109-21.
36. Moseley JB, O'malley K, Petersen NJ, Menke TJ, Brody BA, Kuykendall DH, et al. A controlled trial of arthroscopic surgery for osteoarthritis of the knee. New England Journal of Medicine. 2002;347(2):81-8.
37. Schrøder CP, Skare Ø, Reikerås O, Mowinckel P, Brox JI. Sham surgery versus labral repair or biceps tenodesis for type II SLAP lesions of the shoulder: a three-armed randomised clinical trial. Br J Sports Med. 2017:bjsports-2016-097098.
38. Sihvonen R, Paavola M, Malmivaara A, Itälä A, Joukainen A, Nurmi H, et al. Arthroscopic partial meniscectomy versus sham surgery for a degenerative meniscal tear. New England Journal of Medicine. 2013;369(26):2515-24.

FINAL VERSION OF SIFSO PROTOCOL VERSION 3 Dated 30th June 2022

(finalized and uploaded to clinicaltrials.gov prior to data analysis started)

SacroIliac Joint Fusion vs Sham Operation for treatment of sacroiliac joint pain A prospective double blinded randomized controlled multicenter trial

Engelke Randers, MD

Thomas Johan Kibsgård, MD, PhD

Oslo University Hospital, Department of Orthopaedics, Oslo, Norway

Institute of Medicine, University of Oslo, Oslo, Norway

Paul Gerdhem

Reconstructive Orthopaedics, Karolinska University Hospital, Department of Clinical Sciences, Intervention and Technology, Karolinska Institute, Stockholm, Sweden.

Department of Orthopaedics and Hand Surgery, Uppsala University Hospital and Department of surgical sciences, Uppsala University

Trial registration: The RCT is registered at www.clinicaltrials.gov with identification number NCT03507049

Date of revision: 30th June 2022.

Table of contents:

Introduction…………………………………………………………………..23
Patient and methods…………………………………………………………24
Study design…………………………………………………………...24
Patients…………………………………………………………………...24
Table 1: Inclusion and exclusion criteria…………………25
Interventions…………………………………………………………..26
Figure 1: flow-chart of timeline……………………………27
Sample Size……………………………………………………………28
Outcomes………………………………………………………………28
Study procedures……………………………………………………...30
Data analysis plan……………………………………………………..31
Discussion……………………………………………………………………...38
Ethics, registration and funding…………………………………………...39
Aknowledgements…………………………………………………………..39
References……………………………………………………………………...39

Introduction

Sacroiliac joint (SIJ) pain is increasingly recognized as a possible pain generator in low back pain (LBP) (39). In as many as 15-30% of patients with LBP, the sacroiliac joint may be the cause of pain, and to an even greater extent in patients suffering from “failed back surgery” (1, 39).

The sacroiliac joint transfers force from the spine through the pelvis to the lower extremities (40). Dysfunction of the joint’s articular congruity, ligamentous structures and motor control might be a cause of pain (41). The joint is richly innervated and contains mechano- and nociceptive receptors (42, 43). Even though pain can arise from the sacroiliac joint, the diagnosis of sacroiliac joint pain is challenging. Patient history and radiological imaging have low sensitivity and specificity for the diagnosis of sacroiliac joint pain (3, 44). Single clinical tests show little diagnostic strength for sacroiliac joint pain, but composites of clinical tests have fair sensitivity and specificity (3, 45). To strengthen the diagnosis, clinical tests are combined with intraarticular sacroiliac joint injection with local anesthetics (46).

Sacroiliac joint pain can be severe, disabling and reduce quality of life similarly to other spine conditions (9). Non-operative treatment consists of physiotherapy, pain medication, intra-articular sacroiliac joint steroid injections, prolotherapy and radiofrequency neurotomy of sacral nerve branches. There is limited evidence of effect of these treatment modalities (47-50). In many patients non-operative treatment fails (23). Historically open surgery has often been unsuccessful, due to intense postoperative pain and severe complications (10). Recent multiple minimally-invasive sacroiliac joint fusion studies have reported pain relief and improved function to a greater extent than non-operative treatment and open surgery, and with low frequency of complications (8, 23, 51, 52). The increase in both available implants for minimally invasive sacroiliac joint fusion and their use is demonstrated by a growing number of publications. However, many are industry-sponsored and of poor quality (22, 53-56).

Although the literature suggests superiority of minimally invasive sacroiliac joint fusion to non-operative treatment, some of the effect may be explained by placebo; to what extent is not known. Therefore, we designed a non-industry sponsored prospective multi-center, double-blind, randomized sham-surgery controlled trial (RCT) in accordance with the SPIRIT recommendations to test the null-hypothesis that there is no difference in pain reduction between sham surgery and minimally invasive sacroiliac joint fusion.

Patients and methods

Study design

The trial is designed as a prospective multi-center, double-blinded, randomized sham-surgery controlled trial with 2 parallel groups. Participants, investigators, and data analysisists are blinded for group allocation until the primary endpoint. The primary end-point is group difference in sacroiliac joint pain intensity on the operated side at 6 months postoperatively, measured by Numeric Rating Scale (NRS).

60 participants will be included after oral and written study information is given, and with written consent from the participant. There are 2 recruitment sites; Oslo University Hospital (OUS) in Norway and Karolinska University Hospital in Sweden. The recruitment phase started in April 2018, and the 1st participant was recruited in August 2018 in Norway and December 2019 in Sweden. Due to the corona virus pandemic there were large delays in inclusion in both countries. Inclusion was completed October 2021. All patients are followed for 6 months before unblinding, and for a total of 2 years postoperatively. The studies primary endpoint, the 6 month follow-up was finished by May 2022.

Patients

Referrals from general practitioners and from departments of orthopedic surgery or physical medicine and rehabilitation are screened for eligibility by an orthopedic consultant in the 2 departments and all patients whose referral includes information about pain originating from the sacroiliac joint are taken in for evaluation. This baseline evaluation is performed at the hospital's outpatient clinics with standardized clinical examination in accordance with inclusion and exclusion criteria (Table 1). Those who fulfill the inclusion criteria are invited to participate in the RCT.

Table 1: Inclusion and exclusion criteria

INCLUSION CRITERIA

1. Suspected SIJ pain for >6 months, or >18 months for pregnancy induced pelvic girdle pain.
2. Between 21-70 years old
3. Diagnosis of the SIJ as the suspected primary pain generator based on both of the following:
4. Pain pointed with a single finger (Fortin Finger Test) at or close to the posterior superior iliac spine (PSIS) with possible radiation into buttocks, posterior thigh or groin
5. At least 3 of 6 clinical tests for SIJ pain (3, 45)
    a. Compression
    b. Posterior Pelvic Pain Provocation test – P4
    c. Palpation of the long dorsal sacroiliac ligament
    d. Patrick FABER's test
    e. Active Straight Leg Raise (ASLR) test
    f. Gaenslen´s test
6. Reduced SIJ pain (NRS) of at least 50% of the pre injection NRS score after fluoroscopically or computed tomography guided controlled injection of local anesthetic into the SIJ.
7. Oswestry Disability Index (ODI) score of at least 30%.
8. SIJ pain of at least 5 on the Numeric Rating Scale (NRS), where 0 is no pain at all and 10 is worst imaginable pain.
9. Patient should have tried adequate forms of conservative treatment with little or no response.
10. Bilateral SIJ pain, if one dominant side. If eligible the dominant painful SIJ will be treated in the study.
11. Mentally and physically able to comply with study protocol.
12. Signed study-specific informed consent.

EXCLUSION CRITERIA

1. Pain due to other causes, such as lumbar disc degeneration, lumbar disc herniation, lumbar spondylolisthesis, lumbar spinal stenosis, lumbar facet degeneration, and lumbar vertebral body fracture.
2. Sacroiliac pathology caused by auto-immune disease (e.g. ankylosing spondylitis), neoplasia or crystal arthropathy.
3. History of recent (<1 year) fracture of the pelvis with documented malunion, non-union of sacrum or ilium or any type of internal fixation of the pelvic ring.
4. Spine surgery during the past 12 months.
5. Previously diagnosed or suspected osteoporosis (defined as T-score <-2.5 or history of osteoporotic fracture).
6. Documented osteomalacia or other metabolic bone disease.
7. Any condition or anatomy that makes treatment with the iFuse Implant System infeasible.
8. Patients with prior SIJ surgery.

The orthopedic department at Oslo University Hospital is currently the only center in Norway that performs minimally invasive sacroiliac joint. Approximately 40-60 patients are evaluated per year in the outpatient clinic. Sweden has evaluated twice the number of patients annually, and minimally invasive sacroiliac joint fusion is performed at 2 centers. One of the centers in Sweden participate in the study.

Interventions

Patients are randomized to either receive minimally invasive sacroiliac joint fusion or a sham procedure (Figure 1). All patients will receive the same pre- and postoperative assessment (i.e. blood samples, general anesthesia, draping, wound care and pain medication). They will not be randomized before they are under general anesthesia. Patients are kept under general anesthesia for 40 to 50 minutes regardless of intervention group as this operation on average lasts for 45 minutes. The operations will be done by 1 or 2 surgeons and the theater will be closed to all other personnel. After the procedure is completed the participants will be treated with standard post-operative care as given after minimally invasive sacroiliac joint fusion regardless of randomization group. The post-operative follow-up during the hospital stay will be done by health staff blinded for treatment allocation.

Cases/Surgery

Patients randomized to sacroiliac joint fusion receive treatment with triangular titanium implants according to the surgical technique manual (iFuse®, SI BONE, Inc.) (57). The procedure starts with a 3-5cm long skin incision over the posterolateral aspect of the pelvis. Blunt dissection is performed through the subcutaneous tissue, fascia and muscle. Guide pins are inserted over the SIJ at the desired entry-point, verified by fluoroscopy. The surgeon drills and broaches over the pins, and 3 triangular titanium implants are inserted. The wound is then closed. An injection of the sacroiliac joint with local anaesthetic is performed under guidance of fluoroscopy at the time of the procedure. A subcutaneous injection of local anaesthetic is given around the skin incision.

Controls/Sham surgery

The control group consists of participants randomized to sham operation. The sham operation consists of the surgeon making the same skin incision as the surgical group receives. Blunt dissection is performed through the subcutaneous tissue, fascia and muscle. Guide pins are

Figure 1: Flow-chart of trial timeline.

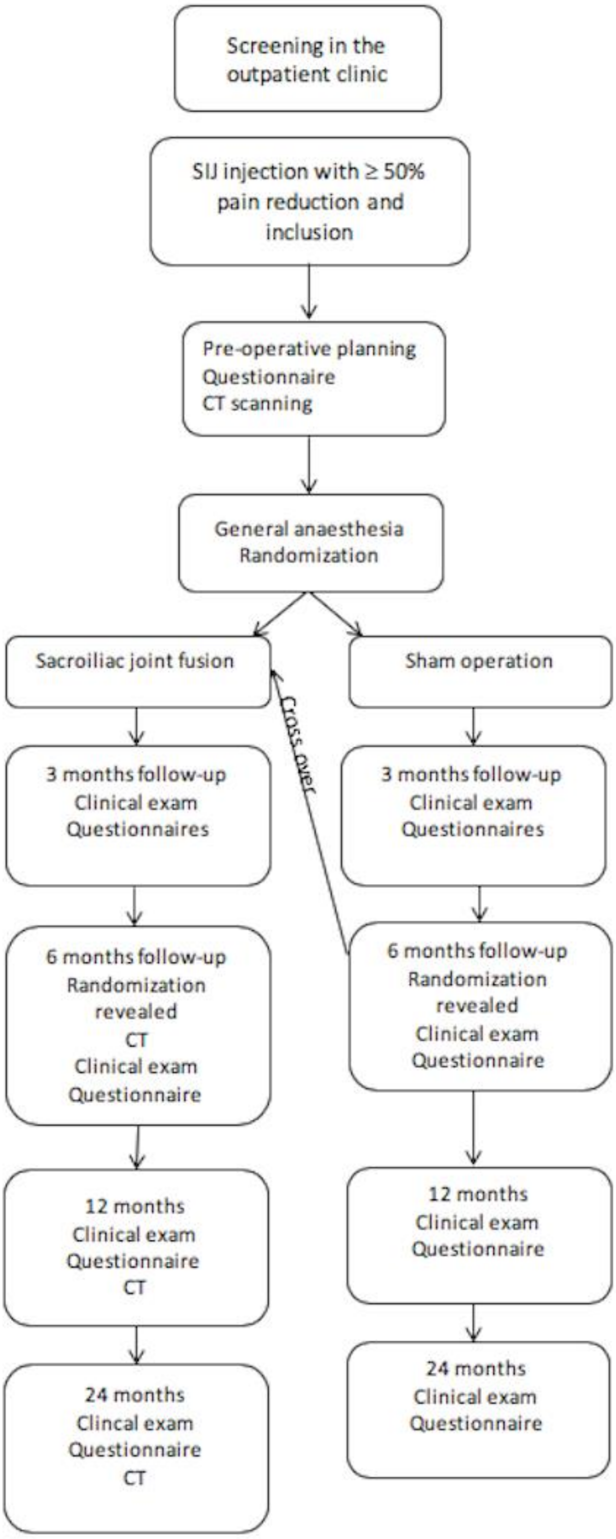

inserted to the ileum, but do not enter the bone. The wound is then closed. Participants will be under general anesthesia while the procedure is simulated. All instruments needed are

smeared with blood from the skin incision in order to keep the 2 procedures as similar as possible. An injection of the sacroiliac joint with local anaesthetic is performed under guidance of fluoroscopy during the procedure. A subcutaneous injection of local anaesthetic is given around the skin incision.
The surgeon will use a standard phrasing for the surgical report for both cases and controls. For controls pre-operative planned implants are reported and for intervention cases the actual implants used are reported.
Initially 2 methods of sham intervention (Norway vs Sweden) were described in the protocol section on ClinicalTrials.gov. The reason for this was due to different criteria for obtaining ethical approval in the 2 countries. However, after observing the 2 different sham intervention methods the study group concluded that the differences where insignificant and therefore could be described similarly.

Sample size

The average mean pre-operative NRS in previous studies has been reported to be 7.0-8.5 (7, 12, 22, 34). There are 2 RCT´s that have compared minimally invasive sacroiliac joint fusion to non-operative treatment (8, 23). These report a mean decrease in global NRS of respectively 4.3 and 5.3 (8, 23). The same 2 studies showed a mean 0.5-1.3 points decrease in NRS in the group treated with non-operative treatment. We assume our trial´s case group to have at least mean 3 points decrease in NRS and the control group to experience a mean 1 point decrease in NRS. 2 previous studies have suggested that 2 points reduction in NRS represents a clinical important difference (32, 33). For the sample size calculation, we assumed a change that would result in a mean 2 point difference between the groups for the main outcome in NRS on the operated side 6 months postoperatively. The standard deviation (SD) was set to 2.5 points since the SD has been reported in previous studies to vary between 1.0-2.9 at 6 months follow-up after sacroiliac joint fusion (7, 12, 22, 34). The probability of a type 1 error (alpha) was set to 0.05. Based on these assumptions we calculate 25 participants in each group with 80% power using an online sample size calculator (http://www.clincalc.com). Due to a possible dropout of 20% we will include 30 participants in each group, giving 60 participants in the study.

<u>Outcomes</u>

Primary outcome

The primary outcome is group difference in pain intensity on the operated side at 6 months postoperatively, measured by NRS, where 0 is no pain at all and 10 is worst imaginable pain. NRS is used as a primary outcome in other studies on minimally invasive sacroiliac joint fusion and will make it possible to compare our results with existing literature (8, 23).

Secondary outcomes

Secondary outcomes are patient-related-outcome-measures (PROMs), functional tests and pain-scores measured with NRS for global pelvic pain, contralateral sacroiliac joint pain and leg pain (Table 2). The PROMs collected are Oswestry Disability Index (58), Pelvic Girdle Questionnaire (29) and the Euroqol 5 dimension (EQ-5D-5L) and EQ visual analogue scale (59). The functional tests consist of the active straight leg raise test, the 6 minute walking test and Timed Up and Go test. Furthermore, data on work status, ambulatory status, adverse events including re-intervention in target sacroiliac joint, implant loosening and fractures are recorded. Patient assessment of treatment and patient satisfaction with treatment is also collected. All assessments at 3- and 6-months follow-up are performed by blinded investigators who are qualified health personnel. After 6 months patients and investigators are un-blinded. Follow-up is continued for 2 years after the operation.

Table 2: Timeline of outcome measures

Endpoint/outcome	Baseline	Preoperative	Postoperative	3 months	6 months	12 months	24 months
NRS operated SIJ/side	x	x	x	x	x	x	x
Global NRS	x	x	x	x	x	x	x
NRS non-operated SIJ/side	x	x	x	x	x	x	x
Leg pain NRS	x	x	x	x	x	x	x
ODI	x	x		x	x	x	x
PGQ	x	x		x	x	x	x
EQ-5D-5L, including EQ-VAS scale	x	x		x	x	x	x
Clinical tests:<br>• Patrick´s test (FABER)<br>• Posterior Pelvic Pain Provocation test (P4)<br>• Gaenslens test<br>• Mennells test<br>• Distraction<br>• Compression<br>• Palpation of the long dorsal ligament and sacrotuberal ligament	x	x		x	x	x	x
Functional tests<br>• ASLR<br>• ASLR range test<br>• 6MWT<br>• TUG	x	x		x	x	x	x
Ambulatory and work status	x	x		x	x	x	x
Adverse Events (including device breakage, migration, loosening etc)			x	x	x	x	x
Re-intervention of target SIJ				x	x	x	x
Patient satisfaction with treatment				x	x	x	x
Patients assessment of treatment				x	x	x	x

Abbreviations and explanations: **SIJ** = Sacroiliac joint. **NRS** = Numeric Rating Scale (measured on scale 0-10 where 0 is no pain and 10 is worst imaginable pain). **ODI** = Oswestry Disability Index (scale 0-100, where 0 is normal function,100 is bedridden). **PGQ** = Pelvic girdle questionnaire (score converted to percentage 0-100%, where 0 is worst pelvic health, 100 is best pelvic health). **EQ-5D-5L**: describes quality of life through scores in 5 dimensions all scored between 1 = no problems to 5= extreme problems. **EQ-VAS** (scale from 0-100: where 0 is worst health and 100 is best health). **ASLR** = Active Straight Leg Raise test (scale 0-5 where 0 = no difficulty raising leg, 5 = severe difficulty. Measured per leg.). **ASLR range test** = Active Straight Leg Raise Range test (measures how high a straight leg can be raised from examination table to the nearest 5 degree). **6MWT** = 6 minute walking test (measures the distance in meters walked in 6 minutes in one round). **TUG** = Timed Up and Go (Measures the time it takes a patient to sit in chair, raise, walk 3 meters back and forth and sit back in chair. Mean time of two rounds is recorded).

Study procedures

Randomization

Participants who give consent to participate in the trial and who fulfill the inclusion criteria will be randomized by the operating surgeon at each site (1 per site) after the participant has been anesthetized. Participants will be randomly assigned to either minimally invasive sacroiliac joint fusion or sham surgery. Randomization is performed as block randomization with blocks of 4 or 6 with a 1:1 allocation to minimally invasive sacroiliac joint fusion or sham surgery. The randomization sequence is pepared by an independent statistician and the randomization sequence was unknown to the researchers. Allocation is completed with a computer generated randomization stratified by site through a specific website (Viedoc

©Viedoc Technologies (60) provided by OUS). This website can only be accessed with personified username and password, and only the operating surgeon has access to randomize participants.

Blinding

All other parties than the operating surgeon and assisting staff in the operating theatre are blinded as to which intervention the participant has received. This includes trial participants, care providers, investigators and data analysts. No other than the operating surgeons have access to randomization prior to follow-up. Un-blinding occurs after completed 6-months follow-up. Participants that have undergone sham intervention and wish to cross over to surgical intervention are allowed to when the 6-month follow-up is completed. Both participants who were randomized to minimally invasive sacroiliac joint fusion initially and participants who cross-over to minimally invasive sacroiliac joint fusion are then followed for at least 2 years postoperatively (Figure 1). Participants who have undergone surgical intervention with implants can undergo surgery of the opposite sacroiliac joint if they wish after the initial 6-month follow-up.

Un-blinding of participants may take place if there is a suspicion of a severe adverse event in which knowledge of implant positioning is essential for patient management. Example of such events can be severe pain, new neurological deficits, or other injuries of such severity that it demands radiological examination to ensure adequate emergency medical care.

Withdrawal

The participant may withdraw from the study for any reason at any time. The investigators may also withdraw participants from the study in order to protect their safety and/or if they are unwilling or unable to comply with the required study procedures.

Postoperative care

All participants are informed of partial weight bearing on crutches for the first 4-8 weeks postoperatively. After 12 weeks there are no restrictions.

CT scan of the sacroiliac joint is performed pre-operatively and at 6, 12, 24 months follow-up to evaluate proper implant placement and lack of radiological signs of loosening.

Data analysis plan

Research questions, objectives and hypotheses

Research questions:

1. What is the efficacy of minimally invasive sacroiliac joint fusion?

2. Are there any tests, signs, symptoms or patient reported outcomes which can predict the outcome of minimally invasive sacroiliac joint fusion?

Objectives:

The main objective of this trial is to test the null-hypothesis that there is no difference in pain intensity between the sham surgery and minimally invasive sacroiliac joint fusion groups at follow-up.

Secondary objectives are to test the secondary hypotheses listed below and investigate the following research questions:

1. Is there a difference in pain intensity between minimally invasive sacroiliac joint fusion and sham at follow-up?
2. Is there a group difference in reduction in pain intensity from baseline to 3 months to 6 months between the sacroiliac joint fusion group and the sham group?
3. Is there a difference in functional outcomes measured with PROMs, clinical tests and functional tests between the sacroiliac joint fusion group and the sham group?
4. Is there a difference in the proportion of individuals that reach a clinically significant improvement of 2 points or more on the NRS scale in the sacroiliac joint fusion group and the sham group?
5. Is there a difference in the usage of analgesics at 3 and 6 months in the sacroiliac joint fusion group and the sham group?
6. Is there a difference in the rate of complications at 3 and 6 months in the sacroiliac joint fusion group and the sham group?
7. Is there a difference in the proportion of individuals that are on full or partial sick leave at 3 and 6 months in the sacroiliac joint fusion group and the sham group?

Hypotheses:

1. There is no difference between the groups in pain intensity on the operated SIJ in the sacroiliac joint fusion group compared to the sham group
2. Pain improves similarly in the sacroiliac joint fusion group as in the sham group between baseline and 3 months.
3. Pain improves similarly in the sacroiliac joint fusion group as in the sham group between 3 months and 6 months.
4. Pain improves similarly in the sacroiliac joint fusion group as in the sham group between baseline and 6 months.

5. There is a similar improvement in physical function, measured with functional tests and subjective questionnaires, in the sacroiliac joint fusion group as in the sham group.
6. The same proportion of individuals in the sacroiliac joint fusion group achieve a clinically significant improvement of 2 points or more on the NRS scale as in the sham group.
7. Individuals in the sacroiliac joint fusion group use the same amount of analgesics at 3 and 6 months as individuals in the sham group.
8. The proportion of individuals with complications at 3 and 6 months is similar in the sacroiliac joint fusion group as in the sham group.
9. The proportion of individuals that are on full or partial sick leave at 3 and 6 months is similar in the sacroiliac joint fusion group as in the sham group

Data collection

Baseline data on age, sex, diagnosis of sacroiliac joint pain, level of sacroiliac joint pain and response to injection if applicable, is registered on all eligible individuals evaluated at the outpatient clinic in order to evaluate reasons for failure to include individuals and for exclusion.

All data from both trial centres is collected into the Viedoc website (©Viedoc Technologies (60)) provided by OUS. The data is confidential and not available as the trial is not completed and recruitment is still ongoing. Data collection is done either by direct electronic registration into the Viedoc Database or by forms, subsequently transcribed into Viedoc by the investigators. If there are missing data these will be collected by contacting the participant or by medical records review. Any adjustment of data entry has to be documented and is registered and reported by the database.

Data set and variables

The data set is exported from the Viedoc database as an excel-file. Only data from baseline, preinjection, postinjection, preoperative, postoperative, 3 months and 6 months time-points are used in this data analysis.

Variables

Independent or Predictor variables		Dependent or outcome variables	
**Continuous**	**Categorical**	**Continuous**	**Categorical**
Age (years)	Sex (F/M)	Numeric Rating Scale (NRS) (0-10 scale):<br>- Operated SIJ<br>- Contralateral SIJ<br>- Leg pain<br>- Global pelvic pain	EQ-5D-5L
Weight (kg)	Etiology of SIJ pain:<br>- Idiopathic<br>- Related to pregnancy<br>- Trauma<br><br>Other	EQ-5D-5L index	
Height (cm)	Civil status		
Clinical tests (pos/neg):<br>- Patrick´s test (FABER)<br>- Posterior Pelvic Pain Provocation test (P4)<br>- Gaenslens test<br>- Mennells test<br>- Distraction test<br>- Compression test<br>- Palpation of Long Dorsal Ligament and Sacrotuberal Ligament.	Employment status		Clinical tests (pos/neg):<br>- Patrick´s test (FABER)<br>- Posterior Pelvic Pain Provocation test (P4)<br>- Gaenslens test<br>- Mennells test<br>- Distraction test<br>- Compression test<br>- Palpation of Long Dorsal Ligament and Sacrotuberal Ligament.
Oswestry Disability Index (%)	Comorbidities (number of previous illnesses)	Oswestry Disability Index (%)	
Pelvic girdle questionnaire (%)	SIJ pain specific questions (Y/N)	Pelvic girdle questionnaire (%)	Adverse Events (Y/N)
EQ-5D VAS scale (0-100)		EQ-5D VAS scale (0-100)	Re-intervention of operated SIJ (Y/N)
Children (number of children)			Patient satisfaction with treatment (0-5)
Timed Up and Go (sec)<br>6 minute walking test (meters)		Timed Up and Go (sec)<br>6 minute walking test (meters)	Patient assessment of treatment (0-5)
Functional tests:<br>- ASLR (0-5)<br>- ASLR range test (0-90degrees)	Pain medication use (No of medication per category)	Functional tests:<br>- ASLR (0-5)<br>ASLR range test (0-90degrees)	Pain medication use (No of medication per category)

Data analysis

Data from the intervention groups will be compared based on the intention to treat (ITT) principle). Statistical comparisons in order to test differences between 2 independent groups will be made by the Student´s t-test or, in the case of a non-normal distribution, the Mann-Whitney U-test. To assess contingency tables, the chi square test will be used or, in the case of small expected frequencies, Fischer´s exact test. Descriptive statistics will be used to characterize the data. A p-value of <0.05 will be considered as significant. A sensitivity analysis will be performed comparing the intention to treat data against the per-protocol data

exclusively from patients who complied with the study protocol. The proportion of patients in both intervention groups who obtain a clinically significant improvement of 2 points and more on the NRS scale will be compared.

In addition, a linear mixed model for repeated measures with a subject-specific random intercept will be used to assess results at the different follow-up time points using the outcome measurements during follow-up as dependent variable and the outcome variable at baseline, time, intervention and the interaction term between time and intervention as fixed effects.

For the primary outcome measure NRS for the operated sacroiliac joint measured at the day of the 6-month follow-up is used.

For the secondary outcomes the baseline value will be taken from the preinjection time point. Where such data is not available at this time point, baseline data will be collected from the preoperative measure point. These baseline values will be compared with values at 3 and 6 month follow-up.

All statistical analysis will be performed by a blinded statistician. The code for group belonging will not be broken until the analyses and interpretations of the results have been performed. Data will be analyzed using IBM SPSS statistical software, version 28.0.1.1 (14). An independent blinded statistician will do additional analyses using STATA statistical software.

Shell tables

1. Demographics

Variable	Intervention group (N=n, CI 95%)	Control group(N=n, CI 95%)
**Age**<br>**20-29:N=**<br>**30-39: N=**<br>**40-49:N=**<br>**50-59: N=**<br>**60-70: N=**	Age groups:<br>20-29:N=<br>30-39: N=<br>40-49:N=<br>50-59: N=<br>60-70: N=	Age groups:<br>20-29:N=<br>30-39: N=<br>40-49:N=<br>50-59: N=<br>60-70: N=
**Sex**<br>- **Female**<br>- **Male**	Female N= / male N=	Female N= / male N=
**Civil status**<br>- **Married**<br>- **Partner**<br>- **Single**		
**Employment status:**<br>- **Employed**<br>- **Sick leave:**<br>○ **Partial (<25%, 25-50%, 50-75%)**<br>○ **Full (100%)**		
**No of Children**		
**BMI and Weight/Height**		
**Ambulatory without assistance**		
**Prior Lumbar fusion**		
**Underlying diagnosis n(%)**<br>- **Pregnancy**<br>- **Trauma**<br>- **Idiopathic**		
**Previous comorbidities (number of)**		
**Taking opioids, n (%)**		
**Preinjection:**<br>- **ODI, score, mean (+/- SD)**<br>- **PGQ, score, mean (+/-SD**<br>- **NRS, score, mean (+/- SD)**<br>○ **operated SIJ**<br>○ **contralateral SIJ**<br>○ **global pelvic pain**<br>○ **leg pain**<br>- **Eq-5D index**		

2. Group difference in NRS sacroiliac joint fusion group versus sham group. Improvement NRS sacroiliac joint fusion group versus sham group. Also demonstrated graphically.

**Months**	**Group difference between surgical group and control group of pain intensity in operated SIJ measured with NRS**
**Baseline (0)**	n/N and mean score, CI 95%
**3months**	n/N and mean score, CI 95%
**6 months**	n/N and mean score, Ci 95%

3. NRS values in sacroiliac joint fusion group compared to sham group. Also demonstrated graphically.

**Months**	**NRS operated SIJ**	
	Surgery	Sham
**Baseline (0)**	n/N and mean score, CI 95%	n/N and mean score, CI 95%
**3months**	n/N and mean score, CI 95%	n/N and mean score, CI95%
**6 months**	n/N and mean score, Ci 95%	n/N and mean score, CI 95%

4. Improvement in NRS shell table proportions

**Months**	**NRS operated SIJ improvement of 2 points or more**		**NRS global pelvic pain improvement of 2 points or more**	
	Surgery	Sham	Surgery	Sham
**Baseline (0)**	n/N and %	n/N and %	n/N and %	n/N and %
**3months**	n/N and %	n/N and %	n/N and %	n/N and %
**6 months**	n/N and %	n/N and %	n/N and %	n/N and %

5. Improvement in ODI and PGQ

**Months**	**ODI**		**PGQ**	
	Surgery	Sham	Surgery	Sham
**Baseline (0)**	mean score, CI 95%	mean score, CI 95%	mean score, CI 95%	mean score, CI 95%
**3months**	mean score, CI 95%	mean score, CI 95%	mean score, CI 95%	mean score, CI 95%
**6 months**	mean score, CI 95%	mean score, CI 95%	mean score, CI 95%	mean score, CI 95%

6. Improvement in TUG and 6MWT

**Months**	**TUG**		**6MWT**	
	Surgery	Sham	Surgery	Sham
**Baseline (0)**	mean score, CI 95%	mean score, CI 95%	mean score, CI 95%	mean score, CI 95%
**3months**	mean score, CI 95%	mean score, CI 95%	mean score, CI 95%	mean score, CI 95%
**6 months**	mean score, CI 95%	mean score, CI 95%	mean score, CI 95%	mean score, CI 95%

7. Shell tables for other outcomes will look and contain similar content as the examples above.

DISCUSSION

Minimally invasive sacroiliac joint fusion is increasingly used worldwide with reported reduced pain, improved physical function and quality of life compared to non-operative treatment in two industry-sponsored RCTs (8, 23). Both showed a clinically and statistically significant difference between the surgically and non-operatively treated groups with greater pain reduction in the surgically treated group (8, 23). It is a weakness that the studies were industry-sponsored. Further, a placebo effect might have contributed to the reported superiority of minimally invasive sacroiliac joint fusion.

To what extent a placebo effect influences the result after minimally invasive sacroiliac joint fusion is not known. A placebo effect after surgery has been shown to be an important factor in efficacy (24). Both patient and health care provider can influence a placebo effect on treatment results. This has particularly been shown in treatment of chronic pain, where the psychological component is believed to be important. Patients with long-lasting diseases seem to be poorer placebo responders, but on the other hand placebo tend to work better in patients expecting to have changes in sensation of pain (26). Several sham controlled studies in orthopedic surgery have shown comparable pain relief in both groups, such as Moseley et al (36, 37). Hence, the effect of minimally invasive sacroiliac joint fusion might be explained by placebo, but this is yet to be examined.

The ethical dilemma of sham surgery is relevant. To ask patients to undergo general anaesthesia for sham surgery might seem unethical. However, a German study of 1,37 million anaesthetic procedures with elective patients, graded as ASA classification physical status I

and II, showed 7,3 cases of death or serious complication per million cases, corresponding to a very low risk (35). All types of surgery are associated with risks of complications, and so also for minimally invasive sacroiliac joint fusion (61). In the control group the risks for surgical complications should be very low. A possible result showing sham surgery to be equal to minimally invasive sacroiliac joint fusion might spare thousands of people from undergoing unnecessary elective surgery with the risk and costs such surgery entail. We therefore find it ethically acceptable to complete a sham-controlled double-blinded randomized prospective study.

Ethics, registration, funding and potential conflicts of interest

Ethical approval for the study has been granted in Norway by the Ethics Committee Health Region Southeast, Oslo, Norway (2017/1892/REK sør-øst A), and in Sweden from The Regional Ethics Committee in Stockholm, Sweden (nr: 2018/1463-31).

The protocol of this study is registered with clinicaltrials.gov (NCT03507049)

The final result from this study will be published in peer-reviewed international journals and also disseminated through international congress lectures.

This trial has received funding from Sophies Minde AS in form of salary payment for the fellow. The Division of Orthopeadic Surgery, Oslo University Hospital is covering the costs of the Norwegian arm of the trial. Metodrådet Stockholm-Gotland covered costs for the ethical consent application in Sweden. Paul Gerdhem is supported by Region Stockholm (for a clinical research appointment) and CIMED, Karolinska Institutet.

None of the principal investigators have any financial or competing interests.

Aknowledgements

We gratefully acknowledge the assistance of the Division of Orthopaedic Surgery at Oslo University Hospital and Karolinska University Hospital for sponsoring and facilitating the trial.

References:

1. DePalma MJ, Ketchum JM, Saullo TR. Etiology of chronic low back pain in patients having undergone lumbar fusion. Pain Med. 2011;12(5):732-9.
2. Simopoulos TT, Manchikanti L, Singh V, Gupta S, Hameed H, Diwan S, et al. A systematic evaluation of prevalence and diagnostic accuracy of sacroiliac joint interventions. Pain Physician. 2012;15(3):E305-E44.
3. Vleeming A, Albert HB, Ostgaard HC, Sturesson B, Stuge B. European guidelines for the diagnosis and treatment of pelvic girdle pain. EurSpine J. 2008;17(6):794-819.
4. Bellamy N, Park W, Rooney PJ. What do we know about the sacroiliac joint? SeminArthritis Rheum. 1983;12(3):282-313.
5. Duhon BS, Cher DJ, Wine KD, Lockstadt H, Kovalsky D, Soo CL. Safety and 6-month

effectiveness of minimally invasive sacroiliac joint fusion: a prospective study. Med Devices (Auckl). 2013;6:219-29.
6. Kibsgård TJ, Røise O, Stuge B. Pelvic joint fusion in patients with severe pelvic girdle pain–a prospective single-subject research design study. BMC musculoskeletal disorders. 2014;15(1):85.
7. Polly DW, Swofford J, Whang PG, Frank CJ, Glaser JA, Limoni RP, et al. Two-Year Outcomes from a Randomized Controlled Trial of Minimally Invasive Sacroiliac Joint Fusion vs. Non-Surgical Management for Sacroiliac Joint Dysfunction. International Journal of Spine Surgery. 2016;10.
8. Sturesson B, Dengler J, Kools D, Pflugmacher R, Prestamburgo D. Sacroiliac minimal invasive fusion compared to physical therapy: six-month outcome from a multicentre randomised controlled trial. The Spine Journal. 2016;16(4):S73-S4.
9. Cher D, Polly D, Berven S. Sacroiliac joint pain: burden of disease. Medical Devices (Auckland, NZ). 2014;7:73.
10. Smith-Petersen MN, Rogers WA. End-Result Study of Arthrodesis of the Sacro-Iliac Joint for ArthritisGÇöTraumatic and Non-Traumatic. The Journal of Bone & Joint Surgery. 1926;8(1):118-36.
11. Hagen R. Pelvic girdle relaxation from an orthopaedic point of view. Acta OrthopScand. 1974;45(4):550-63.
12. Kibsgard TJ, Roise O, Sudmann E, Stuge B. Pelvic joint fusions in patients with chronic pelvic girdle pain: a 23-year follow-up. EurSpine J. 2013;22(4):871-7.
13. Miltner LJ, Lowendorf CS. Low Back Pain: A Study of 525 Cases of Sacro-Iliac and Sacrolumbar Sprain. The Journal of Bone & Joint Surgery. 1931;13(1):16-28.
14. Mitchell JI. Surgical treatment of affections of the lumbo-sacral and sacroiliac joints. Surgery. 1938;4:33-43.
15. Harrison DE, Harrison DD, Troyanovich SJ. The sacroiliac joint: a review of anatomy and biomechanics with clinical implications. JManipulative Physiol Ther. 1997;20(9):607-17.
16. Miller LE, Reckling WC, Block JE. Analysis of postmarket complaints database for the iFuse SI Joint Fusion System(R): a minimally invasive treatment for degenerative sacroiliitis and sacroiliac joint disruption. Med Devices (Auckl). 2013;6:77-84.
17. Al-Khayer A, Hegarty J, Hahn D, Grevitt MP. Percutaneous sacroiliac joint arthrodesis: a novel technique. JSpinal DisordTech. 2008;21(5):359-63.
18. Endres S, Ludwig E. Outcome of distraction interference arthrodesis of the sacroiliac joint for sacroiliac arthritis. Indian J Orthop. 2013;47(5):437-42.
19. Khurana A, Guha AR, Mohanty K, Ahuja S. Percutaneous fusion of the sacroiliac joint with hollow modular anchorage screws: clinical and radiological outcome. JBone Joint SurgBr. 2009;91(5):627-31.
20. Mason LW, Chopra I, Mohanty K. The percutaneous stabilisation of the sacroiliac joint with hollow modular anchorage screws: a prospective outcome study. EurSpine J. 2013;22(10):2325-31.
21. Cummings J, Capobianco RA. Minimally invasive sacroiliac joint fusion: one-year outcomes in 18 patients. AnnSurg InnovRes. 2013;7(1):12.
22. Sachs D, Capobianco R. Minimally invasive sacroiliac joint fusion: one-year outcomes in 40 patients. AdvOrthop. 2013;2013:536128.
23. Polly DW, Cher D, Wine K, Whang P, Frank C, Lockstadt H, et al. Randomized Controlled Trial of Minimally Invasive Sacroiliac Joint Fusion Using Triangular Titanium Implants vs. NonSurgical Management for Sacroiliac Joint Dysfunction. Global Spine Journal. 2016;6(S 01):GP113.
24. Turner JA, Deyo RA, Loeser JD, Von KM, Fordyce WE. The importance of placebo effects in pain treatment and research. JAMA. 1994;271(20):1609-14.
25. Spangfort EV. The lumbar disc herniation. A computer-aided analysis of 2,504 operations. Acta OrthopScandSuppl. 1972;142:1-95.
26. Vandana R, Tushar R. Placebos: current status. Indian journal of pharmacology. 2001;33(6):396-409.
27. Grotle M, Brox JI, Vollestad NK. Concurrent comparison of responsiveness in pain and functional status measurements used for patients with low back pain. Spine (Phila Pa 1976). 2004;29(21):E492-E501.

28. Grotle M, Brox JI, Vollestad NK. Cross-cultural adaptation of the Norwegian versions of the Roland-Morris Disability Questionnaire and the Oswestry Disability Index. JRehabilMed. 2003;35(5):241-7.
29. Stuge B, Garratt A, Krogstad JH, Grotle M. The pelvic girdle questionnaire: a condition-specific instrument for assessing activity limitations and symptoms in people with pelvic girdle pain. PhysTher. 2011;91(7):1096-108.
30. Hollis S, Campbell F. What is meant by intention to treat analysis? Survey of published randomised controlled trials. BMJ. 1999;319(7211):670-4.
31. MacCoun R, Perlmutter S. Blind analysis: Hide results to seek the truth. Nature. 2015;526(7572):187-9.
32. Hagg O, Fritzell P, Nordwall A. The clinical importance of changes in outcome scores after treatment for chronic low back pain. EurSpine J. 2003;12(1):12-20.
33. Farrar JT, Young JP, LaMoreaux L, Werth JL, Poole RM. Clinical importance of changes in chronic pain intensity measured on an 11-point numerical pain rating scale. Pain. 2001;94(2):149-58.
34. Duhon BS, Bitan F, Lockstadt H, Kovalsky D, Cher D, Hillen T, et al. Triangular titanium implants for minimally invasive sacroiliac joint fusion: 2-year follow-up from a prospective multicenter trial. International journal of spine surgery. 2016;10.
35. Schiff J, Welker A, Fohr B, Henn-Beilharz A, Bothner U, Van Aken H, et al. Major incidents and complications in otherwise healthy patients undergoing elective procedures: results based on 1.37 million anaesthetic procedures. British journal of anaesthesia. 2014;113(1):109-21.
36. Moseley JB, O'malley K, Petersen NJ, Menke TJ, Brody BA, Kuykendall DH, et al. A controlled trial of arthroscopic surgery for osteoarthritis of the knee. New England Journal of Medicine. 2002;347(2):81-8.
37. Schrøder CP, Skare Ø, Reikerås O, Mowinckel P, Brox JI. Sham surgery versus labral repair or biceps tenodesis for type II SLAP lesions of the shoulder: a three-armed randomised clinical trial. Br J Sports Med. 2017:bjsports-2016-097098.
38. Sihvonen R, Paavola M, Malmivaara A, Itälä A, Joukainen A, Nurmi H, et al. Arthroscopic partial meniscectomy versus sham surgery for a degenerative meniscal tear. New England Journal of Medicine. 2013;369(26):2515-24.
39. Sembrano JN, Polly DW. How Often Is Low Back Pain Not Coming From the Back? Spine (Phila Pa 1976). 2009;34(1):E27-E32.
40. Vleeming A, Schuenke MD, Masi AT, Carreiro JE, Danneels L, Willard FH. The sacroiliac joint: an overview of its anatomy, function and potential clinical implications. JAnat. 2012;221(6):537-67.
41. Aldabe D, Milosavljevic S, Bussey MD. Is pregnancy related pelvic girdle pain associated with altered kinematic, kinetic and motor control of the pelvis? A systematic review. European Spine Journal. 2012;21(9):1777-87.
42. Szadek KM, Hoogland PV, Zuurmond WW, De Lange JJ, Perez RS. Nociceptive nerve fibers in the sacroiliac joint in humans. Reg AnesthPain Med. 2008;33(1):36-43.
43. Sakamoto N, Yamashita T, Takebayashi T, Sekine M, Ishii S. An Electrophysiologic Study of Mechanoreceptors in the Sacroiliac Joint and Adjacent Tissues. Spine (Phila Pa 1976). 2001;26(20):E468-E71.
44. Elgafy H, Semaan HB, Ebraheim NA, Coombs RJ. Computed tomography findings in patients with sacroiliac pain. ClinOrthopRelat Res. 2001(382):112-8.
45. Laslett M, Aprill CN, McDonald B, Young SB. Diagnosis of sacroiliac joint pain: validity of individual provocation tests and composites of tests. ManTher. 2005;10(3):207-18.
46. Kennedy DJ, Engel A, Kreiner DS, Nampiaparampil D, Duszynski B, MacVicar J. Fluoroscopically Guided Diagnostic and Therapeutic Intra-Articular Sacroiliac Joint Injections: A Systematic Review. Pain Med. 2015;16(8):1500-18.
47. Luukkainen RK, Wennerstrand PV, Kautiainen HH, Sanila MT, Asikainen EL. Efficacy of periarticular corticosteroid treatment of the sacroiliac joint in non-spondylarthropathic patients with chronic low back pain in the region of the sacroiliac joint. Clin Exp Rheumatol. 2002;20(1):52.
48. Cohen SP, Hurley RW, Buckenmaier rCC, Kurihara C, Morlando B, Dragovich A. Randomized

placebo-controlled study evaluating lateral branch radiofrequency denervation for sacroiliac joint pain. Anesthesiology. 2008;109(2):279-88.

49. Patel N, Gross A, Brown L, Gekht G. A Randomized, Placebo-Controlled Study to Assess the Efficacy of Lateral Branch Neurotomy for Chronic Sacroiliac Joint Pain. Pain Med. 2012;13(3):383-98.

50. Kim WM, Lee HG, Jeong CW, Kim CM, Yoon MH. A randomized controlled trial of intra-articular prolotherapy versus steroid injection for sacroiliac joint pain. J Altern Complement Med. 2010;16(12):1285-90.

51. Ledonio CGT, Polly Jr DW, Swiontkowski MF. Minimally Invasive Versus Open Sacroiliac Joint Fusion: Are They Similarly Safe and Effective? Clin Orthop Relat Res. 2014;472(6):1831-8.

52. Whang PG, Darr E, Meyer SC, Kovalsky D, Frank C, Lockstadt H, et al. Long-Term Prospective Clinical And Radiographic Outcomes After Minimally Invasive Lateral Transiliac Sacroiliac Joint Fusion Using Triangular Titanium Implants. Medical Devices: Evidence and Research. 2019;12:411-22.

53. Rappoport LH, Luna IY, Joshua G. Minimally Invasive Sacroiliac Joint Fusion Using a Novel Hydroxyapatite-Coated Screw: Preliminary 1-Year Clinical and Radiographic Results of a 2-Year Prospective Study.(Clinical report). World Neurosurgery. 2017;101:493.

54. Fuchs V, Ruhl B. Distraction arthrodesis of the sacroiliac joint: 2-year results of a descriptive prospective multi-center cohort study in 171 patients. European Spine Journal. 2018;27(1):194-204.

55. Miller LE, Block JE. Minimally invasive arthrodesis for chronic sacroiliac joint dysfunction using the SImmetry SI Joint Fusion system. Med Devices (Auckl). 2014;7:125-30.

56. Sachs D, Capobianco R. One year successful outcomes for novel sacroiliac joint arthrodesis system. AnnSurgInnovRes. 2012;6(1):13.

57. SI BONE. iFuse surgical technique 2020 [Available from: https://si-bone.com/providers/solutions/ifuse/surgical-technique.

58. Copay AG, Daniel JC. Is the Oswestry Disability Index a valid measure of response to sacroiliac joint treatment? Qual Life Res. 2016;25(2):283-92.

59. Foundation ER. EG-5D-5L About 2017 [Available from: https://euroqol.org/eq-5d-instruments/eq-5d-5l-about/.

60. Viedoc. Viedoc 2021 [Available from: https://www.viedoc.com.

61. Shamrock AG, Patel A, Alam M, Shamrock KH, Al Maaieh M. The Safety Profile of Percutaneous Minimally Invasive Sacroiliac Joint Fusion. Global Spine J. 2019;9(8):874-80.

Changes between original protocol and final protocol

1. **Study group**

   The study group altered during the completion of the trial.
   The parties that did not longer contribute exited the trials study group, these were Dr. Jon Dahl and Dr. Andreas Westberg.
   To help with implementation and finalization Professor Lars Nordsletten and Dr. Stephan Rohrl were recruited to the study group.

2. **Change in wording for main aim**

   Both in the first protocol version and initially on clinicaltrials.gov the wording for the main aim was missing "group difference". The main aim was therefore altered to group difference in reduction of NRS in the operated sacroiliac joint both on clinicaltrials.gov (June 2021) and in the final protocol version.

3. **Sham operation**

   The Swedish sham operation was initially described somewhat different from the Norwegian sham operation arm before study start on clinicaltrials.gov.
   When the trial started and the two sham operation variants were observed at the two study sites these were found similar and the description on clinicaltrials.gov was therefore altered to one description for both sites (altered 15 march 2021).

4. **Research questions and objectives:**

   Both research questions and objectives were more clearly defined in the final protocol version.

5. **Variables and shell tables:**

   Variables and shell tables were clearly defined and stated in the final protocol

6. **Functional MRI study arm on clinicaltrials.gov:**

   On clinicaltrials.gov there is one study arm described as a functional MRI study. This functional MRI study is only described in the Swedish ethical approval application protocol and not in the Norwegian (and genereal) final protocol as this arm of the study was only completed on the Swedish participants, as the Norwegian site did not have functional MRI scanning available.

Original Statistical analysis plan dated august 2017:

<u>Statistics</u>

The outcomes (both primary and secondary outcomes) will be analyzed with parametrical statistical methods.

Data from the different groups will be compared based on the 'intention to treat' principle. An intention to treat (ITT) analysis means that all patients, regardless of loss of follow-up or drop-out, remain in the analysis of the group to which they were randomized (30). Multiple imputation of missing data will be used in the ITT analysis. A sensitivity analysis will be performed comparing the ITT data against a per-protocol data exclusively from patients who complied with the study protocol. Continuous and discrete parameters will be measured using parametric or non-parametric tests (depending on skewness) for group comparisons. Categorical parameters will be compared by the Chi-square test.

We will perform blinded statistical analyses (31), in which the individual performing the analyses is unaware of group belonging. The code for group belonging will not be broken until the analyses and interpretations have been performed.

Final Statistical analysis plan dated 30th June 2022:

Data analysis

Data from the intervention groups will be compared based on the intention to treat (ITT) principle). Statistical comparisons in order to test differences between 2 independent groups will be made by the Student´s t-test or, in the case of a non-normal distribution, the Mann-Whitney U-test. To assess contingency tables, the chi square test will be used or, in the case of small expected frequencies, Fischer´s exact test. Descriptive statistics will be used to characterize the data. A p-value of <0.05 will be considered as significant. A sensitivity analysis will be performed comparing the intention to treat data against the per-protocol data exclusively from patients who complied with the study protocol. The proportion of patients in both intervention groups who obtain a clinically significant improvement of 2 points and more on the NRS scale will be compared.

In addition, a linear mixed model for repeated measures with a subject-specific random intercept will be used to assess results at the different follow-up time points using the outcome measurements during follow-up as dependent variable and the outcome variable at baseline, time, intervention and the interaction term between time and intervention as fixed effects.

For the primary outcome measure NRS for the operated sacroiliac joint measured at the day of the 6-month follow-up is used.

For the secondary outcomes the baseline value will be taken from the preinjection time point. Where such data is not available at this time point, baseline data will be collected from the preoperative measure point. These baseline values will be compared with values at 3 and 6 month follow-up.

All statistical analysis will be performed by a blinded statistician. The code for group belonging will not be broken until the analyses and interpretations of the results have been performed. Data will be analyzed using IBM SPSS statistical software, version 28.0.1.1 (14). An independent blinded statistician will do additional analyses using STATA statistical software.

Changes in statistical analysis plan

All over the statistical analysis plan was clearer and more to the point in the last version. Two statisticians, one from Norway and one from Sweden, aided in selecting which statistical analysis methods to use for the data set. The statistical analysis plan was written and defined with their aid.

The main differences were:

1. Shell tables were designed and variables were described prior to statistical analysis.
2. A linear mixed model was included in the analysis plan.
3. Which time-points for outcomes that were to be included in the final data analysis.
